# Role of SPI-1 Secreted Effectors in Acute Bovine Response to *Salmonella enterica* Serovar Typhimurium: A Systems Biology Analysis Approach

**DOI:** 10.1371/journal.pone.0026869

**Published:** 2011-11-11

**Authors:** Sara D. Lawhon, Sangeeta Khare, Carlos A. Rossetti, Robin E. Everts, Cristi L. Galindo, Sarah A. Luciano, Josely F. Figueiredo, Jairo E. S. Nunes, Tamara Gull, George S. Davidson, Kenneth L. Drake, Harold R. Garner, Harris A. Lewin, Andreas J. Bäumler, Leslie Garry Adams

**Affiliations:** 1 Department of Veterinary Pathobiology, College of Veterinary Medicine, Texas A &M University, College Station, Texas, United States of America; 2 Department of Animal Sciences, University of Illinois at Urbana-Champaign, Urbana, Illinois, United States of America; 3 Eugene McDermott Center for Human Growth and Development, The University of Texas Southwestern Medical School, Dallas, Texas, United States of America; 4 Sandia National Laboratories, Computation, Computers and Mathematics Center, Albuquerque, New Mexico, United States of America; 5 Serologix, LLC, Austin, Texas, United States of America; 6 Department of Medical Microbiology and Immunology, School of Medicine, University of California Davis, Davis, California, United States of America; University of Osnabrueck, Germany

## Abstract

*Salmonella enterica* Serovar Typhimurium (*S.* Typhimurium) causes enterocolitis with diarrhea and polymorphonuclear cell (PMN) influx into the intestinal mucosa in humans and calves. The *Salmonella* Type III Secretion System (T3SS) encoded at Pathogenicity Island I translocates *Salmonella* effector proteins SipA, SopA, SopB, SopD, and SopE2 into epithelial cells and is required for induction of diarrhea. These effector proteins act together to induce intestinal fluid secretion and transcription of C-X-C chemokines, recruiting PMNs to the infection site. While individual molecular interactions of the effectors with cultured host cells have been characterized, their combined role in intestinal fluid secretion and inflammation is less understood. We hypothesized that comparison of the bovine intestinal mucosal response to wild type *Salmonella* and a SipA, SopABDE2 effector mutant relative to uninfected bovine ileum would reveal heretofore unidentified diarrhea-associated host cellular pathways. To determine the coordinated effects of these virulence factors, a bovine ligated ileal loop model was used to measure responses to wild type *S.* Typhimurium (WT) and a Δ*sipA*, *sopABDE2* mutant (MUT) across 12 hours of infection using a bovine microarray. Data were analyzed using standard microarray analysis and a dynamic Bayesian network modeling approach (DBN). Both analytical methods confirmed increased expression of immune response genes to *Salmonella* infection and novel gene expression. Gene expression changes mapped to 219 molecular interaction pathways and 1620 gene ontology groups. Bayesian network modeling identified effects of infection on several interrelated signaling pathways including MAPK, Phosphatidylinositol, mTOR, Calcium, Toll-like Receptor, CCR3, Wnt, TGF-β, and Regulation of Actin Cytoskeleton and Apoptosis that were used to model of host-pathogen interactions. Comparison of WT and MUT demonstrated significantly different patterns of host response at early time points of infection (15 minutes, 30 minutes and one hour) within phosphatidylinositol, CCR3, Wnt, and TGF-β signaling pathways and the regulation of actin cytoskeleton pathway.

## Introduction

Despite extensive efforts to understand and prevent *Salmonella* infection, the number of people infected with Salmonellae each year has not changed appreciably in the United States over the last two decades [1]. Approximately 1.4 million people in the United States are infected each year with Salmonellae. This infection is particularly devastating to children and immunocompromised adults. In the United States, children under the age of 4 are disproportionately affected by salmonellosis (72 cases per 100,000 children) [2]. A recent study estimates that there are annually 93.8 million cases of *Salmonella* gastroenteritis worldwide, resulting in 155,000 deaths [3]. In parts of Africa where HIV infection is widespread, Non-typhoidal *Salmonella* (NTS) infection is recognized as a major health problem and is the leading cause of pediatric bacteremia [4], [5], [6]. In severe cases, particularly in immunocompromised individuals, Salmonellae penetrate the intestinal mucosa and enter the bloodstream leading to systemic infection. Understanding the acute phase of intestinal infection is important to designing intervention strategies for children and immunocompromised adults where CD4^+^ T cell help, which is required to prevent disseminated infection, may be diminished or absent.

Calves are ideal models for human *Salmonella* infection, because they develop diarrhea with clinical and pathologic features similar to those in people [7], [8], [9], [10]. In both people and calves, salmonellosis is characterized by influx of neutrophils into the intestine and fluid secretion into the intestinal lumen, accompanied by severe gastroenteritis with abdominal cramping and diarrhea. During the first few hours of intestinal infection, polymorphonuclear cells (PMNs) leave the vasculature and migrate across the mucosal barrier, and ultimately arrive at the lumen of the intestine [9]. Increased vascular permeability is associated with PMN migration and movement of fluid from the circulation into the intestinal lumen characterizes the first 8 to 12 hours of infection. This movement of fluid may form the basis for the diarrhea exhibited during enteric salmonellosis [9]. Influx of PMNs and fluid secretion into the intestinal lumen are hallmarks of *Salmonella*-induced enteritis in both humans and cattle. These naturally occurring clinical and pathological similarities between human and bovine infection make the calf an ideal animal to model the clinical signs of salmonellae infection in humans [8], [11], [12].

*Salmonella enterica* Serovar Typhimurium (*S.* Typhimurium) is one of the top *Salmonella* serotypes that cause disease in humans and cattle. Invasion of the intestinal epithelium is critical for the establishment of infection and occurs within the first 15 minutes of bacterial contact [9], [13], [14]. *S.* Typhimurium invades the non-phagocytic enterocytes of the host intestinal epithelium through manipulation of the host actin cytoskeleton by *Salmonella* effector proteins secreted through the Type III secretion system (T3SS-1) encoded at *Salmonella* Pathogenicity Island I [13], [15], [16], [17], [18], [19]. *Salmonella* effector proteins bind to the actin cytoskeleton and induce the formation of cell membrane ruffles that facilitate *Salmonella* internalization [20], [21], [22], [23], [24], [25], [26], [27], [28], [29], [30], [31].

Beyond invasion, the T3SS-1 delivered effector proteins are required for PMN migration and fluid secretion in the intestine. Previous studies have shown that PMN migration and fluid secretion in bovine intestinal loop model are the result of the coordinated actions of five effector proteins secreted by T3SS-1: SipA, SopA, SopB, SopD, and SopE2 [12], [32]. Recent studies demonstrate the individual functions of these effector proteins: SipA binds actin directly; SopA is a HECT E3 ubiquitin ligase; SopB is a multi-functional phosphatidyinositol phosphatase with roles in actin cytoskeleton signaling, apoptosis, and trafficking of the *Salmonella* containing vacuole (SCV); SopE2 activates actin cytoskeleton rearrangements through Rho family GTPases; and SopD participates in invasion and has a role in membrane fission and macropinosome formation [29], [33], [34], [35], [36], [37], [38], [39], [40]. Individual loss of the genes encoding these proteins only partially decreases fluid secretion in the bovine intestine relative to that induced by wild type *S.* Typhimurium. Loss of all five genes together (Δ*sipA*, *sopABDE2*) significantly diminishes fluid secretion, PMN influx, and lesions in the intestine [12]. Further, SipA, SopA, SopB, SopD, and SopE/E2 have combined roles in C-X-C chemokine expression [12], [32]. Loss of all five genes decreases host expression of the genes encoding C-X-C chemokines, GROα, GROγ, interleukin 8 (IL-8), and CXCL6 (GCP-2) [32]. The CXC chemokines are chemotactic for PMNs and induce their migration from the vasculature to sites of infection. Therefore, the increased expression of these genes may in part explain PMN migration to the intestine. It is well known that T3SS-1 secreted effectors SipA, SopA, SopB, SopD and SopE2 are required for *Salmonella* induced bovine and human diarrhea, however, the host cellular pathways and pathway mechanistic genes critical to fluid secretion, PMN transmigration, enteritis and diarrhea are less well defined. We hypothesized that comparison of the response of bovine intestinal mucosa to wild type *Salmonella* and a SipA, SopABDE2 effector mutant relative to uninfected bovine ileum would reveal heretofore unidentified diarrhea-associated host cellular pathways. In addition, we also hypothesized that this comparison would lead to the discovery of specific pathway mechanistic genes. To test our hypothesis, we challenged bovine ligated ileal loops containing Peyer's patch with three conditions; wild type *Salmonella* Typhimurium, an isogenic SipA, SopA, SopB, SopD and SopE2 deletion mutant, or media as a negative control. We measured the host mucosal gene expression profile sequentially for 12 hours post-inoculation. We then compared the data from the host response to the three conditions by conventional and dynamic Bayesian analytical methods, confirming known cellular pathways associated with salmonellosis and identifying additional cellular pathways. Known and newly discovered host cellular pathways were merged to establish functional models of the host∶pathogen interface and to identify pathway mechanistic genes for future study. In this study, we report the comparison of host responses to wild type *Salmonella* as compared to the SipA, SopABDE2 mutant demonstrating significantly different patterns and perturbations of host cellular pathways at early time points of infection and disease expression relative to the inflammatory response and diarrhea.

## Materials and Methods

### Bacterial Strains and Culture

Derivatives of *Salmonella enterica* serotype Typhimurium strain IR715 and ZA21, were used in this study. IR715, a derivative of ATCC 14028, is a spontaneous nalidixic acid resistant derivative referred to hereafter as “wild type” (WT) and ZA21 is a previously described Δ*sipA sopABDE2* mutant (MUT) [12]. Cultures were grown in Luria-Bertani (LB) broth supplemented with the following antibiotics as appropriate at the indicated concentrations: nalidixic acid (50 mg/l), kanamycin (100 mg/l), chloramphenicol (30 mg/l), and tetracycline (20 mg/l). To prepare the bacterial inoculum, IR715 and ZA21 were cultured in LB broth for 18 hours at 37°C with shaking at 150 rpm in a shaking incubator (Model C24, New Brunswick Scientific, Edison, NJ). The resultant cultures were diluted 1∶100 in sterile LB broth and incubated as before for an additional 4 hours. Bacteria in the exponential phase of growth were harvested by centrifugation for 15 min at 1,500× *g* and re-suspended in fresh LB broth to obtain a final concentration of approximately 0.75×10^9^ colony-forming units/ml (CFU/ml). The bacterial concentration of the inoculum was confirmed by spreading serial 10-fold dilutions on agar plates containing the appropriate antibiotics.

### Animals

Four male Holstein calves, 4–6 weeks of age and weighing 45–55 kg, were used in each experiment under an approved animal use protocol in accordance with animal use policy under the supervision of the Texas A & M University Institutional Animal Care and Use Committee (IACUC). All animal work was approved under Animal Use Protocols 2004-029 and 2004-037. Calves were obtained from a local, commercial source and were unrelated. The calves were fed antibiotic-free milk replacer twice daily and water *ad libitum*. To minimize the possibility of interference from other enteropathogens on the host gene expression profile, calves were tested twice for fecal excretion of *Salmonella* spp. and *Eimeria* spp. oocysts and only animals that tested negative for these bacteria and parasites were used. Fecal specimens and rectal swabs were collected from calves two weeks prior to and immediately before the experiment and cultured for the presence of *Salmonella* serotypes. Swabs were placed in tetrathionate broth (BBL, Sparks, MD) overnight and subsequently plated onto XLT-4 agar plates (BBL). Fecal flotation with a hypertonic saline solution was used for detection of *Eimeria* spp. oocysts. All four calves were clinically healthy before the experiment and were culture negative for fecal excretion of *Salmonella*. We elected to use four calves because previously published work in our lab indicated that four calves would be sufficient to detect significant differences between the treatments used in this study [32].

### Bovine Ligated Ileal Loop Surgeries

The bovine ligated ileal loop surgical procedure has been described previously [41]. Briefly, the calves were fasted for 12 hours prior to the surgery. Anesthesia was induced with propofol (Abbot Laboratories, Chicago, IL) followed by placement of an endotracheal tube and maintenance with isoflurane (Isoflo, Abbot Laboratories) for the duration of the experiment. A laparotomy was performed, the distal jejunum and ileum containing Peyer's patches were exposed, and 21 loops approximately 6 cm in length were ligated with umbilical tape, leaving 1 cm loops between them. Overnight bacterial cultures were grown as described above. The ileal loops were inoculated by intralumenal injection of 3 ml of a suspension of either *S.* Typhimurium strain IR715 (WT) or *ΔsipA sopABDE2* mutant (MUT) in LB broth containing approximately 0.75×10^9^ CFU/ml. Loops injected with sterile LB broth (3 ml) served as uninfected, negative controls (UI). Loops were alternately inoculated with LB broth, WT, or MUT starting at the loop closest to the ileocecal junction and were subsequently excised in the same order as inoculated. Three loops were removed at each time point, one loop for each condition. Each loop was inoculated with only one bacterial strain or the negative control. The loops were replaced into the abdominal cavity until collection by excision. At each of seven time points (15 min, 30 min, 1, 2, 4, 8, and 12 hours), three loops (one LB inoculated control loop, one wild type inoculated loop, and one mutant inoculated loop) were excised. From each of these loops, samples were collected for histopathology, bacteriology, electron microscopy, frozen sections, and RNA extraction.

### Morphologic Analysis

For morphologic analysis by light microscopy, full cross-sections of each loop (including the Peyer's patch) were fixed in formalin, processed according to the standard procedures for paraffin embedding, sectioned at 5 µm thickness, stained with hematoxylin and eosin, and examined by light microscopy. All tissue sections collected contained Peyer's patch, which is continuous for approximately 1.2 meter proximal to the ileocecal junction in the calf. Previous work has demonstrated that ligated ileal loops constructed in the manner described have virtually identical morphology and cell composition [9].

### Quantification of Tissue-associated S. Typhimurium

Two-6 mm biopsy punches (0.1 g) from the anti-mesenteric side of the intestinal mucosa to maximize Peyer's patch collection were excised from every loop, washed three times in PBS to remove extracellular bacteria, homogenized, and diluted in 1 ml of PBS. Similar procedures were followed for the control loops to identify the presence of viable *Salmonella* as a consequence of leakage of the loops, cross contamination during material processing, or *Salmonella* migration via lymphatic or blood vessels. To determine the number of viable CFU of *S.* Typhimurium in tissues, lysates were serially diluted and cultured on LB agar with nalidixic acid (50 mg/l). Bacterial counts represent tissue-associated bacteria and do not distinguish between intracellular and extracellular bacteria. For RNA isolation, only the intestinal mucosa and submucosa were collected, but for bacteriology, the tissues were full thickness intestinal tissue containing the mucosa, submucosa, and smooth muscle layers.

### Statistical Analysis of Bacteriology

For analysis of invasion percentages, the data were transformed logarithmically. The geometric means were determined, and the statistical significance of differences was calculated using two-tailed Student's *t* test.

### RNA Extraction

Total RNA was extracted after dissection of the ileal loops obtained at 15 min and 30 min and at 1, 2, 4, 8, and 12 hours post-infection. A 6 mm biopsy punch was used to collect 8 tissue pieces of Peyer's patch from the excised loop. The mucosa and submucosa of the samples were immediately dissected, the tissue pieces finely minced with a sterile scalpel blade, and two biopsy punches (0.1 mg of tissue) transferred to 1 ml of Trizol reagent (Molecular Research Center, Cincinnati, OH). Tissues were immediately further disrupted with an RNase, DNase free plastic disposable pestle. The RNA extraction was performed using the recommended protocol from the manufacturer. The resultant RNA pellet was re-suspended in DEPC-treated water (Ambion, Austin, TX). Genomic DNA was removed by RNase-free DNase I treatment (DNA-free, Ambion, Austin, TX) according to the manufacturer's instructions, and samples were stored at −80°C until used.

### RNA Sample Analysis

The quality of the RNA was initially assessed by measurement of the λ260/280 ratio and agarose gel electrophoresis. RNA concentration was quantified by measuring absorbance at λ260 nm using a NanoDrop® ND-1000 (NanoDrop, Wilmington, DE), and the RNA quality was determined using a Nano-Chip® on an Agilent 2100 Bioanalyzer (Agilent, Palo Alto, CA). Reference RNAs (rat liver total RNA and rat liver mRNA) were included on each of the four chips used for analysis. The RNA analyzed in all samples was of good to excellent quality (results not shown). The average RNA integrity number (RIN) was 7.42 and the median was 7.7 (Range 6.1–8.8 with the exception of four samples that had RIN values between 2.5 and 3.4). Samples were characterized by distinct 18S and 28S rRNA peaks and RNA size distribution. Ten micrograms of RNA was used as starting material for each array.

### Preparation of Bovine Reference RNA

A reference RNA was used to normalize signal intensity across all arrays [42], [43], [44], [45]. This method was used rather than co-hybridizing treatment and control samples to the same array to allow comparison of bovine intestinal response not only to *Salmonella* but also to other pathogens, including *Brucella melitensis*, *Mycobacterium avium* subsp. *paratuberculosis*. Each of these pathogens cause significant disease in ruminants and potentially humans. Comparison of intestinal host response to these pathogens was a first step in building a model of intestinal host pathway interactions with the intent of building an *in silico* interactome model. These studies and description of the interactome model will be described elsewhere. A reference RNA was chosen over genomic DNA as reference RNA is subjected to the same procedures, such as reverse transcription, as sample RNA thereby providing control for RNA or dye degradation during processing. Total RNA was isolated from Madin-Darby bovine kidney (MDBK) and bovine B lymphocyte (BL-3) cell lines (American Type Culture Collection, Manassas, VA) and fresh bovine cerebral cortex and cerebellum collected from surgery calves following euthanasia at the conclusion of each experiment. RNA from this combination of tissues was experimentally shown to produce cDNA that would hybridize to the majority of the open reading frames (ORFs) represented on the microarray [46]. Cell lines were grown in 150 cm^2^ cell culture flasks with Eagle's minimum essential medium (E-MEM) (ATCC) supplemented with 10% heat-inactivated fetal bovine serum (HI-FBS). Tissues were homogenized in ice-cold Tri-reagent® (Ambion). RNA concentration and quality from each sample was determined before and after pooling the samples. Total RNA isolated from three samples was pooled together in equal amounts, aliquoted and stored at −80°C until needed.

### Construction and Annotation of Bovine cDNA Microarrays

A custom, bovine cDNA array consisting of 13,257 unique 70-mer oligonucleotides representing 12,220 cattle ORFs was designed from normalized and subtracted cattle cDNA libraries. The oligos were printed in 150 mM phosphate buffer at 20 µM concentration in duplicate on aminosilane-coated glass slides at the W. M. Keck Center (University of Illinois at Urbana-Champaign). The oligos were annotated based on the GenBank accession number, when available. Further description of the microarray and its development has been previously published [47], [48]. The gene array annotation was based on bovine Unigene Build #95.

### Microarray Sample Preparation and Slide Hybridization

Microarrays were used to examine the transcriptional profiles of bovine intestinal epithelia (control and wild type or mutant infected) across seven time points (15 min, 30 min, 1, 2, 4, 8, and 12 hours). Experiments were performed in quadruplicate (four animals), generating a total of 84 slides with the array printed in duplicate on each slide. The labeling and hybridization procedures have been described previously [47]. Briefly, cDNA from bovine experimental samples (i.e. from infected and control loops) and cDNA generated from the bovine reference RNA sample were co-hybridized to the previously described custom 13K bovine 70-mer oligoarray. The cDNA was reverse-transcribed using Superscript III reverse transcriptase (Invitrogen, Carlsbad, CA) and labeled with amino-allyl-UTP (Ambion, Austin, TX). The cDNA generated from the bovine reference RNA was labeled with Cy3 while the cDNA generated from the RNA samples isolated from the intestinal loops was labeled with Cy5. Cy3 and Cy5 dye esters were covalently linked to the amino-allyl group by incubating the samples with the dye esters in 0.1 M sodium carbonate buffer. Prior to hybridization, the microarrays were denatured by exposing to steam from boiling water for three seconds, UV cross-linked and then immersed in pre-hybridization buffer [5X sodium chloride, sodium citrate buffer (SSC), 0.1% sodium dodecyl sulfate (SDS) (Ambion, Austin, TX), 1% bovine serum albumin (BSA)] at 42°C for a minimum of 45 min. The arrays were then washed four times in RNase-, DNase-free, distilled water, immersed in 100% isopropanol for 10 seconds, and dried by centrifugation. Slides were hybridized at 42°C for approximately 40 hours in a dark, humid chamber (Corning, Corning, NY), washed for 10 min at 42°C with low stringency buffer [1 X SSC, 0.2% SDS] and then washed twice for 5 min each wash in a higher stringency buffer [0.1 X SSC, 0.2% SDS and 0.1 X SSC] at room temperature with agitation.

### Microarray Data Acquisition and Analysis

Immediately after washing, the slides were scanned using a commercial laser scanner (GenePix 4100; Axon Instruments Inc., Foster City, CA). The spots representing genes on the arrays were adjusted for background and normalized to internal controls using image analysis software (GenePix Pro 4.0; Axon Instruments Inc.). Spots with fluorescent signal values below background were disregarded in all analyses. Samples were normalized against the bovine reference RNA signals across slides and within each slide (across duplicate spots). Regression analysis was performed to verify reproducibility of replicate arrays, all of which had an R^2^ value of 0.70 or greater. This R^2^ value may have resulted from poor RNA quality in a small number of the arrays (predominantly arrays generated from the 12 hour time point). Pairwise comparisons and Student's *t* test with Benjamini-Hochberg correction were performed using GeneSifter (VizX Labs, Seattle, WA), and Spotfire DecisionSite 9.0 (Spotfire, Inc., Somerville, MA). Genes were considered to be significantly altered if the average fold-change was at least 1.5, the adjusted *p* value was less than 0.05, the alteration was reproducible across replicates, and the magnitude of difference between conditions was greater than 50% of the fold-change obtained for any two replicate controls. Replicate spots, representing a single gene were also expected to “agree.”

Comparative pathogenicity analysis and modeling was performed using an integrated platform termed the BioSignature Discovery System (BioSignatureDS™) (Seralogix, LLC, Austin, TX). This approach for genomic data analysis and modeling at the system biology level offers an integrated view of biological mechanisms and networks of interactions. Specifically for the analysis reported herein, the tools were used to: 1) determine significant gene modulations via a statistical inference *z*-score method employing a Bayesian estimate of variance [49] thresholding technique and fold-change; 2) conduct biological system level analysis employing dynamic Bayesian network models for scoring and ranking of metabolic pathways, signaling pathways and gene ontology (GO) groups; 3) conduct dynamic Bayesian candidate mechanistic gene analysis to identify genes within the network models that are most responsible for causing pathway and GO group perturbations; and 4) create a genetic network system model derived from the candidate mechanistic genes and their genetic interactions. Description of the computational techniques employed by BioSignatureDS™ for the analyses and modeling of the data reported herein is described in File S1. Individual gene *z*-scores and fold-change data are provided in Tables S1 and S2 respectively.

### Microarray Data Deposited in the Gene Expression Omnibus

All microarray data is MIAME compliant and the original images and extracted data were deposited in the Gene Expression Omnibus at the National Center for Biotechnology Information (http://www.ncbi.nlm.nih.gov/geo/), Accession #GSE13847.

### Microarray Results Validation

Ten selected bovine genes with differential expression by microarray results were analyzed by quantitative RT-PCR (qRT-PCR). Two micrograms of RNA samples were reverse-transcribed using TaqMan® Reverse Transcription Reagents (Applied Biosystems). For relative quantification of target cDNA, samples were either run in individual tubes in SmartCycler II (Cepheid, Sunnyvale, CA) using one SmartMix bead (Cepheid) for 2–25 µl PCR reaction along with 20 ng of cDNA, 0.2X SYBR Green I dye (Invitrogen) or run using 20 ng of cDNA in Sybr Green PCR Master Mix (Applied Biosystems) on an ABI 7500 Real-time PCR System according to the manufacturer's instructions. In either case, Primer Express Software v2.0 (Applied Biosystems) was used to design forward and reverse primers that were used at a concentration of 0.3 µM (Table 1). For each gene tested, the individual calculated threshold cycles (C_T_) were averaged among each condition and normalized to the C_T_ of the bovine GAPDH, from the same cDNA samples before calculating the fold-change using the ΔΔC_T_ method [50]. For each primer pair, a negative control (water) and an RNA sample without reverse transcriptase (to determine genomic DNA contamination) were included as controls during cDNA quantification.

**Table 1:** Primer sequences for real time PCR validation of microarray results.

GENBANK ACCESSION	GENE SYMBOL	GENE NAME	FORWARD PRIMERS (5′-3′)	REVERSE PRIMERS (5′-3′)	REF
NM_001001175	Apoc3	Apolipoprotein C3	GCACCAAGACCGCCAA	GCAGATTCCCAGAAGTCAGTGAA	This work
NM_174006	CCL2	Chemokine (C-C motif) ligand 2	TCCTAAAGAGGCTGTGATTTTCAA	AGGGAAAGCCGGAAGAACAC	[79]
NM_174007	CCL8	Chemokine (C-C motif) ligand 8	CCAAAGCGGACAGGGATGT	GTCCAGGAGCCTTATGGAAGTCT	This work
NM_173878	F3	Coagulation Factor III (F3)	TCTTGCGATTAGCATCTTGGTTT	ATGGTTTGATGTCAGCCTCTCA	This work
NM_182786	FOS	v-fos FBJ murine osteosarcoma viral oncogene homolog	ATCTGCTGAAGGAGAAGGAAAAAC	TGAGGAGTGGCAGGGTGAAG	This work
NM_001034034	GAPDH	Glyceraldehide-3-phosphate dehydrogenase	TTCTGGCAAAGTGGACATCGT	GCCTTGACTGTGCCGTTGA	[32]
NM_173923	IL6	Interleukin 6	AAACCGAAGCTCTCATTAAGCG	TGGAAGCATCCGTCCTTTTC	This work
NM_175793	MAPK1	Mitochondrial-activated protein kinase 1	GGCTTGGCCCGTGTTG	GGAAGATGGGCCTGTTGGA	[79]
NM_174576	PIK3R2	phosphoinositide-3-kinase, regulatory subunit 2 (p85 beta)	GCTCCTCTACCCAGTGTCCAAGT	ACGCTGTCCTCCTTGACGAT	This work
NM_174147	PLAU	plasminogen activator, urokinase	TGAACGGAGGAAAATGTGTAACC	TCAGAAGGACGGTGGGAGAG	This work
NM_174198	TLR4	Toll-like Receptor 4	AATGGCAGGCAACTCTTTTCA	GGGCTACCTGTTCCAGTTGACA	This work

## Results

### Salmonella Invasion and Inflammation in the Intestinal Epithelium

**Figure 1:**
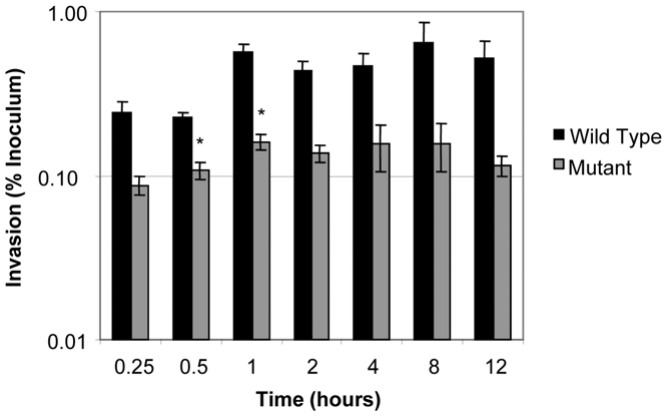
Invasion of bovine intestinal mucosa by *S. enterica* serovar Typhimurium. Data are plotted as the geometric means from four independent assays. The bars represent the standard errors. The percent invasion normalized to the inoculum for wild type *S.* Typhimurium is represented by the black bars and the Δ*sipA*, *sopABDE2* mutant is represented by the grey bars. Asterisks indicate a significant difference in invasion compared with the wild type positive control (*p*≤0.05).

Four male, unrelated, *Salmonella* culture negative calves underwent ligated ileal loop surgery. Loops in each calf were inoculated with wild type *S.* Typhimurium (WT), a Δ*sipA*, *sopABDE2* mutant (MUT), or sterile culture medium as a negative control (UI). Individual loops from each treatment were collected at seven time points (0.25, 0.5, 1, 2, 4, 8, and 12 hours) post inoculation. Each intestinal loop contained Peyer's patch which in the calf extends approximately 1.2 meters cranial from the ileocecal junction. The morphologic description of the ligated ileal loops and *Salmonella* infection in the calf intestine has been previously described [9]. Loops were consistent in cellular composition and response to infection throughout the length of intestine used. Prior to analyzing gene expression, bacterial invasion and uptake by M cells and phagocytes were assessed by bacterial counts measuring tissue-associated bacteria and analyzing morphologic changes in the tissue (Figure 1 and data not shown). There was a small but significant difference in bacterial numbers between WT and MUT infected loops at 30 minutes and 1 hour post inoculation (Figure 1). This difference may have resulted from the invasion defect that has been previously described for this mutant. There were no differences at later time points which could result from either extracellular bacteria or uptake of the mutant by M cells or other cells. Consistent with previous reports, the MUT inoculated loops had significant reductions in PMN migration into the intestinal mucosa and submucosa, inflammation score, and fluid secretion relative to the WT inoculated loops (data not shown) [32].

### Transcriptional Profile of Bovine Host Peyer's Patches Inoculated with S. Typhimurium

RNA was isolated from the mucosa and submucosa of the Peyer's patch of each intestinal loop. There were a total of 84 RNA samples that were used to generate indirectly labeled cDNA. The cDNAs were co-hybridized on a custom bovine microarray with indirectly labeled cDNA generated from a bovine reference RNA. The same bovine reference RNA was used throughout the experiment and was used for normalization of the microarrays. Gene expression changes associated with infection by WT and the MUT were compared with gene expression in the uninfected loops (UI) and with each other.

Two methods of microarray data analysis were employed to determine gene expression changes: Student's *t* test with Benjamini & Hochberg (B&H) multiple hypothesis testing and a statistical inference *z*-score method employing a Bayesian estimate of variance [49], [51]. Multiple hypothesis testing provides an index for defining the expected proportion of false positives (false discovery rate, FDR) among all significance tests to identify a set of “candidate positives,” of which a high proportion are likely to be true. This *t* test employed a B&H-corrected *p* value of <0.05 (95% confidence) combined with a |fold-change| of 1.5 as the cutoff values for significance testing. Likewise, the Bayesian statistical inference method employed a |*z*-score| >1.96 (95% confidence) combined with a |fold-change| >2 as the threshold values for inference testing. Whereas significance testing is a case of *post-hoc* analysis, Bayesian inference generates probabilistic statements about hypotheses based on the microarray data (*a priori* estimation) [49]. Significance testing and Bayesian inference identified respectively 732 and 1046 genes that exhibited significant changes in expression in WT loops relative to UI loops (Table 2 and 3 and detailed gene lists in Tables S4 and S5). In MUT loops there were 203 and 637 significantly altered genes as measured by significance testing and Bayesian statistical inference, respectively (Table 2 and 3 and Tables S4 and S5). According to the Bayesian statistical inference analysis, there were 983 genes significantly altered (uniquely different) between WT and MUT (Table 3 and Table S4). A surprising number of transcripts represented genes of unknown function. Within the 1046 altered genes identified by Bayesian inference, there were 444 transcripts of unknown function in WT loops (Table S4). Within the 637 altered genes identified by Bayesian inference, there were 300 transcripts of unknown function in MUT loops (Table S4). Significance of gene expression changes was consistent (i.e. overlapped) between significance testing and Bayesian inference in 348 genes in WT loops and only 11 genes in MUT loops (Table 4 and Table S5). We propose that the data normalization processing steps and the Bayesian variance estimation technique are the source for not having a higher number of overlapping altered genes between the two analysis methods. The Bayesian inference method employed: 1) a less stringent spot quality filtering technique; 2) a more sophisticated universal reference normalization method in conjunction with Lowess correction; and 3) a Bayesian variance estimator that infers a better prediction of the standard deviation for genes which have a low number of biological replicates. Overall, the Bayesian statistical inference method identified a larger number of altered genes than the *t* test alone [49].

**Table 2:** Summary of significant genes determined by *t* test using cutoff *p* value ≤0.05 and |fold-change| ≥1.5 in response to infection with wild type *Salmonella* Typhimurium or an isogenic Δ*sipA*, *sopABDE2* mutant.

Condition	Total Number Changed	Upregulated/Downregulated	Total Number of Genes	Time post inoculation (hours)						
				0.25	.5	1	2	4	8	12
WT loops only	732	Up-regulated	495	14	5	265	23	45	80	204
		Down-regulated	251	19	9	1194	9	3	65	77
MUT loops only	203	Up-regulated	39	0	4	2	0	0	8	27
		Down-regulated	164	143	1	2	0	0	1	17
Genes with common regulation between conditions	24	Up-regulated	18	0	0	1	0	0	1	17
		Down-regulated	6	1	0	1	0	0	1	3
Genes that differed between WT and MUT	732	Up-regulated	496	14	5	264	23	44	79	187
		Down-regulated	250	18	9	118	9	3	64	74

WT loops = wild type inoculated loops; MUT loops = Δ*sipA*, *sopABDE2* mutant inoculated loops. Detailed gene list is provided in Table S3. Total number of changed genes is the unique set of altered genes across all time points and regulation direction. Genes with common regulation are defined by the intersection of the sets of genes found significant for WT and for MUT conditions, whereas the genes that differ are the set of significant genes in WT that are not found in MUT condition.

**Table 3:** Summary of significant genes determined by Bayesian inference *z*-score using cutoff |*z*-score| ≥1.96 and |fold-change| ≥1.5 in response to infection with wild type *Salmonella* Typhimurium or an isogenic Δ*sipA*, *sopABDE2* mutant.

Condition	Total Number Changed	Upregulated/Downregulated	Total Number of Genes	Time post inoculation (hours)						
				0.25	0.5	1	2	4	8	12
WT loops only	1046	Up-regulated	670	23	63	252	41	147	268	310
		Down-regulated	493	20	9	350	57	71	53	169
MUT loops only	637	Up-regulated	574	161	139	33	163	120	116	304
		Down-regulated	165	10	28	16	50	16	39	41
Genes with common regulation between conditions	202	Up-regulated	167	2	17	8	25	49	95	114
		Down-regulated	40	0	1	9	8	0	9	18
Genes that differed between WT and MUT	983	Up-regulated	589	21	46	244	16	98	173	196
		Down-regulated	488	20	8	341	49	71	44	151

WT loops = wild type inoculated loops; MUT loops = Δ*sipA*, *sopABDE2* mutant inoculated loops; Detailed gene list is provided in Table S4. Total number of changed genes is the unique set of altered genes across all time points and regulation direction. Genes with common regulation are defined by the intersection of the sets of genes found significant for WT and for MUT conditions, whereas the genes that differ are the set of significant genes in WT that are not found in MUT condition.

**Table 4:** Summary of significantly altered genes that overlap between the two statistical methods (Table 2 and 3).

Condition	Total Number Changed	Upregulated/Downregulated	Total Number of Genes	Time post inoculation (hours)						
				0.25	0.5	1	2	4	8	12
WT loops only	348	Up-regulated	228	3	4	92	8	31	69	118
		Down-regulated	111	2	2	84	3	1	7	28
MUT loops only	11	Up-regulated	10	0	1	0	0	0	1	8
		Down-regulated	1	1	0	0	0	0	0	0
Genes with common regulation between conditions	6	Up-regulated	6	0	0	0	0	0	0	6
		Down-regulated	0	0	0	0	0	0	0	0
Genes that differed between WT and MUT	335	Up-regulated	228	3	4	92	8	31	69	112
		Down-regulated	111	2	2	84	3	1	7	28

Genes with significant *z*-score change and significant *p* value and 1.5-fold-change in response to infection with wild type *Salmonella* Typhimurium or an isogenic Δ*sipA*, *sopABDE2* mutant.

WT loops = wild type inoculated loops; MUT loops = Δ*sipA*, *sopABDE2* mutant inoculated loops; Detailed gene list is provided in Table S5. For genes to be on this list, they had to be found significant based on both statistical analysis methods (*t* test and Bayesian statistical inference). Genes with common regulation are defined by the intersection of the sets of genes found significant for WT and for MUT conditions, whereas the genes that differ are the set of significant genes in WT that are not found in MUT condition.

### Temporal Differences in Gene Expression

**Figure 2:**
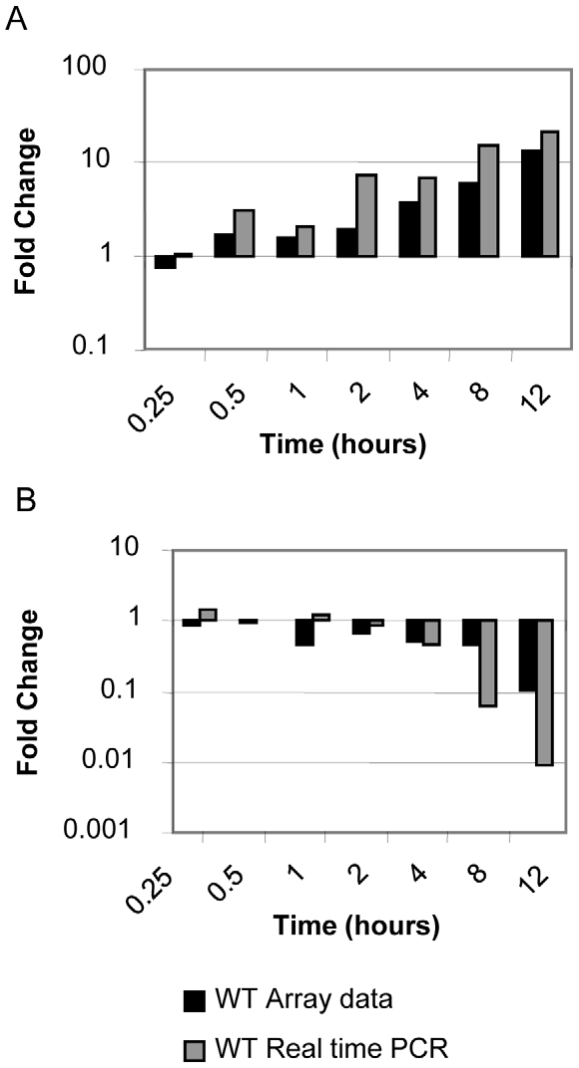
Comparison of gene expression analysis by microarray with quantitative real time PCR. Microarray (black bars) and qRT-PCR (gray bars) fold-changes for both an up-regulated gene, *CCL2* (A) and a down-regulated gene, *ApoC3* (B) are represented. The means of 4 independent assays using RNA from ligated ileal loops inoculated with wild type *Salmonella* Typhimurium (IR715), both microarrays and qRT-PCR, performed in duplicate are shown.

**Figure 3:**
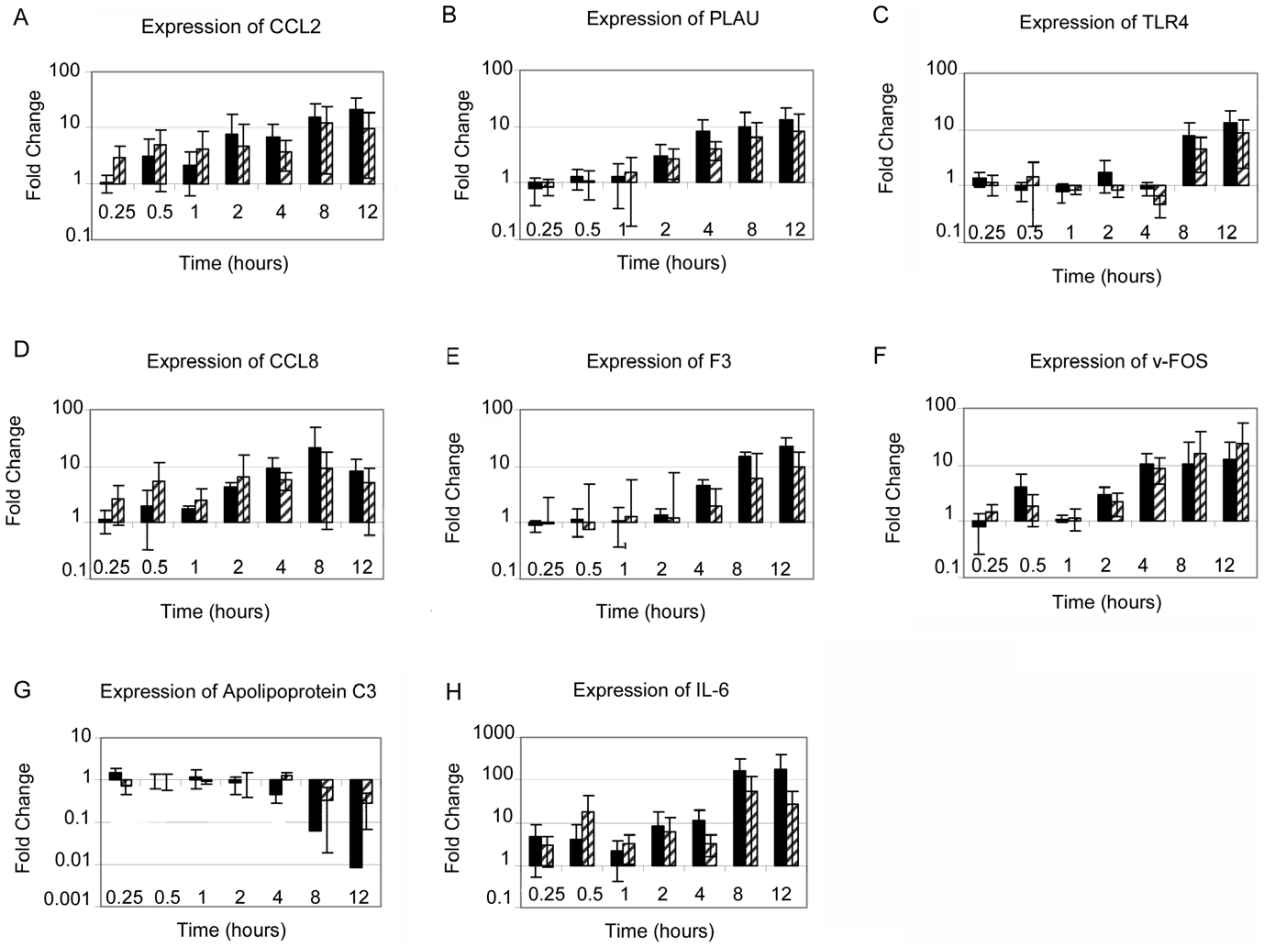
Gene expression changes in wild type *S. enterica* serovar Typhimurium and Δ*sipA, sopABDE2* mutant inoculated ileal loops measured by quantitative real time PCR. Real time PCR data were calculated using the Δ (Δ) CT method with wild type and mutant compared to negative control loops. Glyceraldehyde 3 phosphate dehydrogenase (GAPDH) was used as the housekeeping gene for normalization. Black bars represent wild type *S.* Typhimurium. Gray bars represent the ΔsipA, sopABDE2 mutant. Expression was measured for CCL2 (A), PLAU (B), TLR4 (C), CCL8 (D), F3 (E), v-FOS (F), Apoliporotein C3 (G), and IL-6 (H). Means +/- Standard Deviation (error bars) of 4 independent assays performed in duplicate are shown.

Temporally, WT infection induced a biphasic host response with a period of intense gene expression activity at one hour followed by a second phase at later time points of progressively increased activity over time. In MUT loops there was no biphasic response. The number of significantly affected genes increased progressively over time with a pattern of primarily increased gene expression at later time points (Tables 2A and B). While there was substantial overlap between gene expression patterns exhibited by the WT loops and MUT loops, there were significant differences between the WT and MUT at individual time points (Tables S4 and S5). As shown by the representative examples in Figure 2, gene expression changes were consistent between microarray and qRT-PCR for both up-regulated and down-regulated genes. Microarray gene expression data were validated by qRT-PCR. Six genes determined to be significantly affected in the host response to infection by both methods of analysis (significance testing and Bayesian methods) were chosen for verification (Figure 3). In addition to these genes, two genes identified by Bayesian inference as significantly regulated, the genes encoding apolipoprotein C3 and interleukin 6, were also tested by qRT-PCR and found to be significantly regulated by that method as well (Figure 3G and H). This was true for all of the genes tested by qRT-PCR. Differences in gene expression due to infection, either with WT or MUT, that were detected by the microarray were consistent with differences detected by qRT-PCR both for genes with increased expression and genes with decreased expression relative to the negative control (Figure 3 and data not shown). Additionally, 602 genes were represented on the microarray with more than one probe. In general, multiple probes for the same gene showed consistent expression between the probes. Probes with inconsistent regulation are noted where significant.

### Regulation of Chemokine Expression

Chemokines are signaling molecules responsible for recruitment of leukocytes. Previous work demonstrated increased expression of C-X-C chemokines during *Salmonella* infection in the bovine intestine [9], [32], [52], [53]. For additional confirmation of microarray results, gene expression patterns from previously published work were compared to the expression patterns in the data presented here and were found to be consistent. In particular, increased expression of C-C motif and C-X-C motif chemokines including *CCL2*, *CCL8*, *CXCL6 (GCP2)*, and *IL8* (*CXCL8*), and their receptors including *CCR1* and *CCR3* was seen in both WT and MUT inoculated loops at 4, 8, and 12 hours (Table 5). Inclusion in this table required a |*z*-score| ≥2.24 at any one time and reflects 97.5% confidence in the data (note that no fold-change cutoff was applied). The magnitude of expression of these genes was greater in WT than in MUT, but there was no significant difference between these conditions. Expression of *IL8*, *IL8RB*, *CXCL6*, and *CCL8* differed between WT and MUT at 8 hours between these conditions (Table 5). Increased gene expression of three additional chemokines, *CXCL9*, *CXCL10*, and *CXCL11* was also seen in both WT and MUT infection but did not differ between the two conditions (Table 5). These genes encode chemokines induced by interferon-γ (IFN-γ) and Type I interferons (Interferon-α and Interferon-β). These chemokines are chemotactic for T cells through the chemokine receptor CXCR3. IFN-γ is produced in response to *Salmonella* infection and is encoded by *IFNG* which was strongly induced in both WT and MUT loops in this study consistent with previously published results. Additional genes that were compared and consistent with previously published data in calves included *TNF*, *IL1B*, *IL1RN*, and *IL17*.

**Table 5:** Chemokine gene expression *z*-score.

Treatment	Symbol	Description	Probe	t = 15	t = 30	t = 60	t = 120	t = 240	t = 480
WT	CCL2	chemokine (C-C motif) ligand 2	BF043950	−1.25	1.44	0.86	0.85	4.77	8.81
MUT				2.06	2.45	0.73	1.46	3.47	8.08
W vs M				−1.49	−0.26	0.78	0.18	1.07	1.81
WT	CCL8	chemokine (C-C motif) ligand 8	NM_174007	0.25	0.35	0.11	1.98	3.87	7.22
MUT				2.77	2.47	1.38	2.87	2.52	5.76
W vs M				−1.54	−0.80	−0.34	0.32	1.46	2.63
WT	CXCL6	chemokine (C-X-C motif) ligand 6 (granulocyte chemotactic protein 2)	NM_174300	−0.72	1.32	1.29	2.12	4.58	7.18
MUT				0.30	2.22	0.53	1.84	4.38	5.75
W vs M				−1.11	−0.38	1.18	0.64	0.30	2.50
WT	CXCL9	chemokine (C-X-C motif) ligand 9	BM362452	−1.01	0.25	0.38	−0.07	−0.06	2.46
MUT				−0.51	1.00	−0.25	0.57	0.03	2.91
W vs M				−0.45	−0.44	0.80	−0.3	0.33	0.64
WT	CXCL10	chemokine (C-X-C motif) ligand 10	CK955893	0.51	1.04	−0.27	0.54	3.45	7.96
MUT				1.57	1.10	−0.24	0.74	2.44	6.98
W vs M				−1.07	0.26	0.74	−0.04	0.70	0.12
WT	CXCL11	chemokine (C-X-C motif) ligand 11	CK959898	−0.32	0.24	1.29	0.02	1.91	3.13
MUT				0.01	0.82	−0.29	0.31	1.71	4.15
W vs M				−0.39	−0.19	1.41	0.53	0.28	−0.01
WT	IL8	interleukin 8	BF039564	−0.29	0.64	0.68	2.06	5.89	9.35
MUT				0.95	3.97	1.20	2.72	4.55	5.80
W vs M				−0.79	−0.91	0.16	0.31	0.67	3.06
WT	IL8RB	interleukin 8 receptor, beta	NM_174360	0.40	0.01	−0.19	0.61	1.55	4.45
MUT				1.43	0.51	0.65	0.89	1.23	1.23
W vs M				−0.33	−0.01	−0.36	0.05	0.66	2.60

WT = Comparison of gene expression in wild type inoculated loop versus uninfected control loops.

MUT = Comparison of gene expression in Δ*sipA*, *sopABDE2* mutant inoculated loops versus uninfected control loops.

W vs M = Comparison between wild type inoculated loops with Δ*sipA*, *sopABDE2* mutant inoculated loops.

Probe numbers are GenBank reference numbers.

t = time in minutes.

### Dynamic Bayesian Modeling Analysis of Pathway Activation

**Figure 4:**
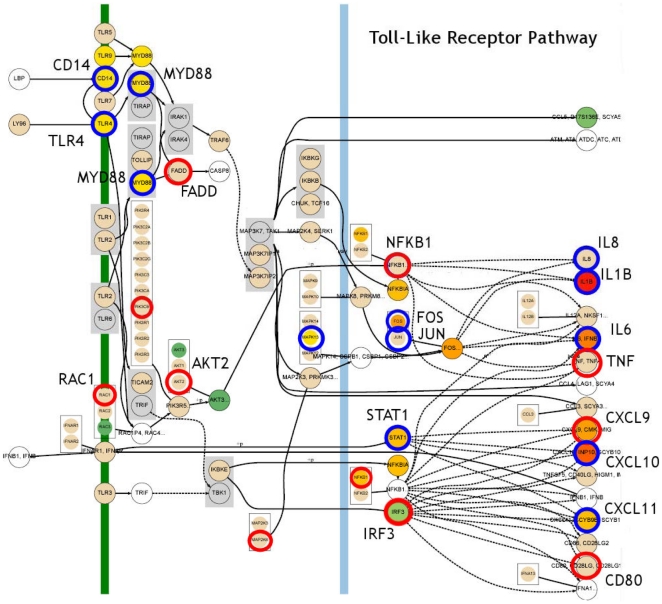
Wild type *S. enterica* serovar Typhimurium Toll-like Receptor Model with Mechanistic Genes. This is an example of the DBN model showing the mechanistic genes indicated by concentric rings around the node. The network model reveals the parent and child relationships and the state of gene expression for any selected time point. Increasing up regulation is indicated by the gradient colors from light yellow to dark red and down regulation is from light green to dark green. The red rings indicate mechanistic genes unique to the WT condition, while the blue rings indicate those in common with the MUT condition.

To better understand the complex molecular interactions between host and pathogen, gene expression data were mapped to 219 molecular interaction pathways using the Dynamic Bayesian Gene Group Activation (DBGGA) method described more fully in File S1. The DBGGA method is similar to Bayesian network modeling methods employed by other investigators [54], [55], [56], [57], [58], [59], [60]. DBGGA creates a Dynamic Bayesian network (DBN) model for each pathway based on KEGG [61], [62] while a naïve Bayesian classifier [63] type network model is created for each GO category. Employing DBGGA, significantly activated pathways under both WT and MUT conditions in comparison to uninfected controls were identified and a differential comparison between WT and MUT was made. As an example, the WT *S.* Typhimurium modeling result for the Toll-like Receptor Pathway is shown in Figure 4 where the discovered mechanistic genes are indicated by concentric rings around the node. In this figure, the red rings indicate mechanistic genes that are unique to the WT condition while the blue rings are mechanistic genes in common between the WT and MUT conditions. The graphical model is used to view the state at any time point of all measured genes upstream and downstream of each mechanistic gene candidate. The products of mechanistic genes control key regulatory points in pathways through a variety of methods including altering gene transcription or translation or through post-translation processes such as protein phosphorylation. Alteration of mechanistic genes induces substantial effects on biological processes. See File S1 for the full mechanistic gene definition found significant from DBGGA Bayesian *z*-score method.

**Figure 5:**
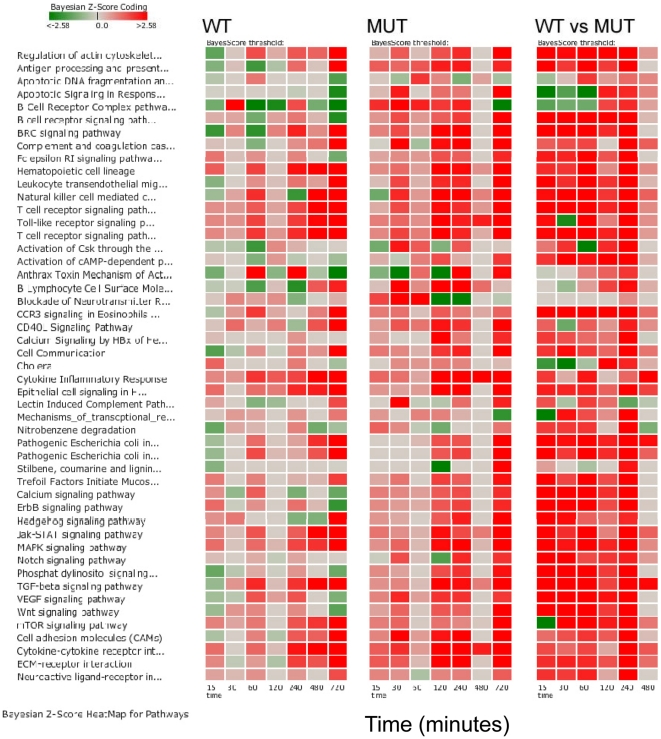
Pathway scores in bovine intestinal loops inoculated with wild type *S. enterica* serovar Typhimurium and the Δ*sipA*, *sopABDE2* mutant. Heat map comparison of pathway scores for each host condition by sampling time point post infection was generated. Pathways were limited to the categories of cell mobility, cell communication, cell growth and death, infectious diseases, immune system, membrane transport, and signal transduction were compared. The score magnitudes are shown as a gradient color from light to bright red for induced and from light to bright green for suppressed pathway activity. Comparisons between wild type inoculated loops and control loops are labeled WT; between the Δ*sipA*, *sopABDE2* mutant inoculated loops and control loops inoculated with LB broth are labeled MUT; and between wild type and the Δ*sipA*, *sopABDE2* mutant inoculated loops are labeled WT vs. MUT.

There were 193 pathways that were significantly altered between WT and MUT infected loops (WT vs MUT) at various time points throughout the experiment (Table 6). Pathways within the categories of cell mobility, cell communication, cell growth and death, infectious diseases, immune system, membrane transport, and signal transduction were compared. There were 49 pathways represented in these categories (Figure 5). Note that WT versus MUT compares the WT expression values to the MUT expression values and the MUT is considered the control condition. Within the 49 perturbed pathways (either activated or repressed) were several that have been extensively studied in *Salmonella* infection including Regulation of Actin Cytoskeleton, Apoptosis [64], [65], and the Toll-like receptor (TLR) signaling pathway [66], [67]. A temporal pattern of activation or repression for these pathways was generated (Figure 5). These 49 pathways are proposed to represent target pathways for intervention in *Salmonella*-induced diarrhea.

**Table 6:** Pathways that differed in regulation between early and late time points in WT vs MUT based on Bayesian *z*-score described in Table S2.

Name	Description	t = 15	t = 30	t = 60	t = 120	t = 240	t = 480
hsa00300	Lysine biosynthesis	0.57	2.19	1.95	−2.47	2.45	0
hsa00380	Tryptophan metabolism	2.13	2.01	1.77	1.14	2.54	0
hsa00220	Urea cycle and metabolism of amino groups	1.93	0.73	1.41	1.2	2.97	0
hsa00902	Monoterpenoid biosynthesis	0.35	0.37	0.54	0	2.81	−0.89
hsa00900	Terpenoid biosynthesis	−1.15	0.14	1.57	2.52	2.4	0
hsa01031	Glycan structures - biosynthesis 2	1.99	1.4	1.49	0.53	2.5	0
hsa00531	Glycosaminoglycan degradation	1.87	1.84	0.79	1.44	3.36	0.96
hsa04610	Complement and coagulation cascades	1.44	1.63	1.7	0	2.93	1.45
hsa99107	B Lymphocyte Cell Surface Molecules pathway	0	−0.69	0.64	1.75	2.42	0.42
hsa99111	Calcium Signaling by HBx of Hepatitis B virus pathway	1.85	1.58	1.25	1.1	2.59	−0.11
hsa05120	Epithelial cell signaling in Helicobacter pylori infection	2.19	2.1	2.17	1.41	2.85	1.75
hsa00626	Nitrobenzene degradation	1.84	1.86	1.91	0.16	2.65	−1.08
hsa00940	Stilbene, coumarine and lignin biosynthesis	0.62	0	−0.5	0	2.3	0
hsa00591	Linoleic acid metabolism	0.57	−0.78	1.56	0.44	2.37	0
hsa04940	Type I diabetes mellitus	1.68	1.66	−1.95	0.51	3.09	2
hsa00730	Thiamine metabolism	1.8	1.19	−1.14	2.12	3.33	0
hsa00410	beta-Alanine metabolism	1.9	2.1	1.35	−0.85	2.68	0
hsa04514	Cell adhesion molecules (CAMs)	1.22	1.58	1.91	1.49	3.21	0.28
hsa04060	Cytokine-cytokine receptor interaction	1.68	1.27	1.72	0.49	2.82	1.52
hsa04512	ECM-receptor interaction	1.99	2.17	2.09	1.6	3.47	1.23
hsa04080	Neuroactive ligand-receptor interaction	1.95	1.82	2.15	0.52	3.18	0
hsa00641	3-Chloroacrylic acid degradation	−1.27	−1.05	−1.05	0.41	3.28	0
hsa00362	Benzoate degradation via hydroxylation	0.22	1.42	−1.8	3.17	2.29	0
hsa99104	Apoptotic Signaling in Response to DNA Damage pathway	−3.68	−1.57	−3.09	2.19	2.09	0.94
hsa04330	Notch signaling pathway	2.67	2.57	1.69	1.9	2.18	0.67
hsa04340	Hedgehog signaling pathway	2.55	2.43	1.38	1.11	2.06	0.08

WT = Comparison of gene expression in wild type inoculated loop versus uninfected control loops.

MUT = Comparison of gene expression in Δ*sipA*, *sopABDE2* mutant inoculated loops versus uninfected control loops.

The *z*-score >|2.24| reflects 97.5% confidence in the data.

t = time in minutes.

**Figure 6:**
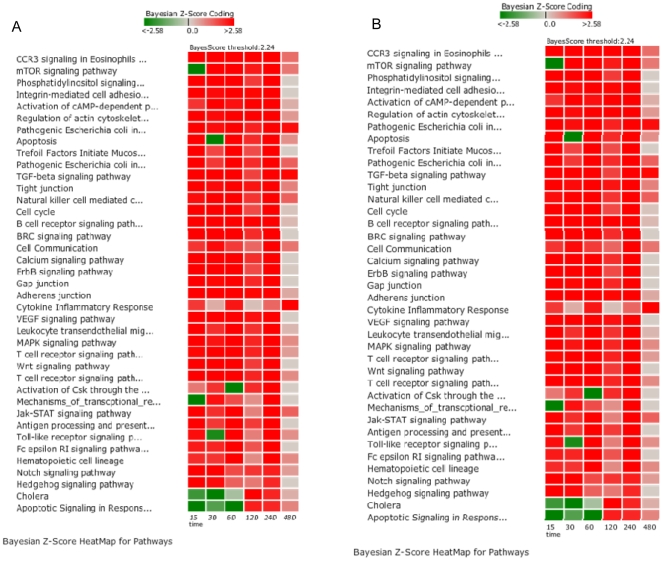
Temporal differences between pathways scores for wild type *S. enterica* serovar Typhimurium and the Δ*sipA*, *sopABDE2* mutant. Pathway activation in wild type and mutant inoculated loops was compared and evaluated for temporal changes. Pathways activated in early time points (15 minutes, 30 minutes, and 60 minutes) were summarized (A) and pathways activated in late time points (120, 240, and 480 minutes) were summarized (B). Pathways were limited to the categories of cell mobility, cell communication, cell growth and death, infectious diseases, immune system, membrane transport, and signal transduction. The score magnitudes are shown as a gradient color from light to bright red for induced and from light to bright green for suppressed pathway activity.

**Figure 7:**
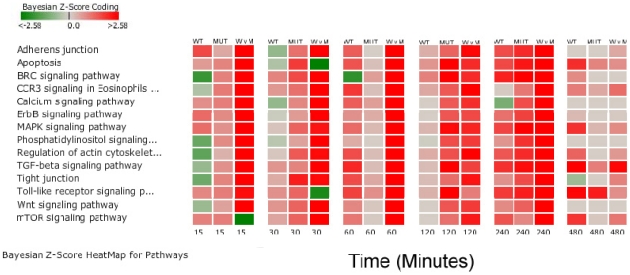
Temporal differences within a subset of pathways that differentiate infection with wild type *S. enterica* serovar Typhimurium and a Δ*sipA*, *sopABDE2* mutant. Temporal changes in pathway activation in wild type and mutant inoculated loops were evaluated in a subset of pathways. Pathways were limited to the categories of cell mobility, cell communication, cell growth and death, infectious diseases, immune system, membrane transport, and signal transduction. The score magnitudes are shown as a gradient color from light to bright red for induced and from light to bright green for suppressed pathway activity. Comparisons between wild type inoculated loops and control loops are labeled WT; between the Δ*sipA*, *sopABDE2* mutant inoculated loops and control loops inoculated with LB broth are labeled MUT; and between wild type and the Δ*sipA*, *sopABDE2* mutant inoculated loops are labeled W v M.

**Figure 8:**
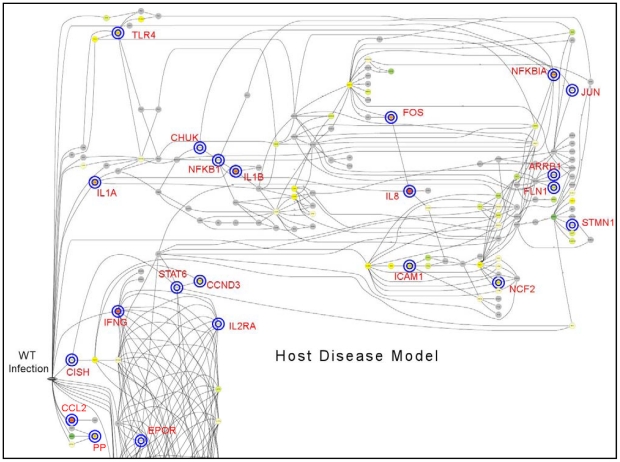
Dynamic Bayesian Network Disease Model of wild type *S. enterica* serovar Typhimurium. A host disease model was constructed by combining the network structures of the top ten scoring pathways for WT *S.* Typhimurium. The key mechanistic genes are labeled with their common gene names. The blue rings indicated mechanistic genes (|Bayesian *z*-score| >2.24) as derived from the scoring described for the Dynamic Bayesian Gene Group Activation technique (File S1).

Because a temporal pattern of increased gene activity at one hour was previously noted in WT loops, differences between WT and MUT loops at early time points (15 minutes, 30 minutes, 1 hour) and late time points (2 hours, 4 hours, and 8 hours) were explored in greater depth (Tables 2 and 3). The 12 hour time point was not evaluated because of reduced signal quality in the microarrays from the 12 hour time points. The pathways that differed in significant regulation between early and late time points at a minimum of at least one time point are summarized in Table 6. Pathways within the categories of cell mobility, cell communication, cell growth and death, infectious diseases, immune system, membrane transport, and signal transduction were compared for differences between WT and MUT in early and late time points (Figure 6). Within this pathway sub-group, Apoptotic Signaling in Response to DNA Damage, Hedgehog Signaling and Notch Signaling differentiated WT from MUT loops at early time points. Multiple pathways differentiated the two conditions at late time points including the Complement and Coagulation Cascades, Cytokine Receptor Signaling, and Epithelial Cell Signaling in *Helicobacter pylori* (Table 6; Figure 6). At eight hours, there were very few pathways that were significantly different between WT and MUT. To explore differences between WT and MUT further, a set of the significantly affected pathways within the subgroup was investigated to identify key regulatory genes that differed in expression during early infection (Figure 7). Pathways with primary functions in metabolism were not considered in this group. This subset included 1) Adherens Junction; 2) Apoptosis; 3) BRC signaling; 4) CCR3 Signaling in Eosinophils; 5) Calcium Signaling; 6) ErbB Signaling; 7) MAPK Signaling; 8) Phosphatidylinositol Signaling System; 9) Regulation of Actin Cytoskeleton; 10) TGF-β Signaling Pathway; 11) Tight Junction; 12) Toll-like Receptor Signaling; 13) Wnt Signaling and; 14) mTOR Signaling pathways. The changes in activation status between WT and UI, MUT and UI, and WT and MUT over time can be seen in Figure 7. The inter-relationship between genes in these pathways is summarized in the host disease model in Figure 8. The method for implementing the disease (system) model is described in File S1.

All 14 of the pathways exhibited activation in the MUT loops at 2 hours, 4 hours, and 12 hours. This was not the case in WT inoculated loops where the pattern was more complex. At 15 minutes post-infection, Phosphatidylinositol Signaling System, Regulation of Actin Cytoskeleton, Tight Junction, Wnt Signaling, TGF-β Signaling, BRC Signaling, and CCR3 Signaling in Eosinophils pathways were down-regulated. By one hour, CCR3 Signaling, Regulation of Actin Cytoskeleton, TGF-β Signaling, and Tight Junctions were all activated. These differences between WT and MUT are consistent with differential regulation of host response through the five deleted effector proteins. These seven pathways are connected through regulation of the actin cytoskeleton and phosphatidylinositol signaling and despite early down-regulation, by one hour all but BRC Signaling, Wnt Signaling and Phosphatidylinositol Signaling System were activated. Within the 14 selected pathways, there were 103 genes that were common key mechanistic genes within the pathways. Of these genes, 11 were identified as key intersection points having mechanistic functions in more than five of the 14 pathways (Table S7). These mechanistic genes are *AKT2*, *JUN*, *MAPK1*, *NFKB1*, *NFKB2*, *PIK3C2G*, *PIK3C3*, *PIK3R2*, *PRKCG*, *RAC1*, and *RHOA*. Based on Bayesian *z*-score, only *AKT2*, *RHOA*, and *NFKB1* at 1 hour were appreciably regulated (|Bayesian *z*-score| >2.24). None of these genes were significantly modulated at later time points, however small alterations in expression of these key genes can have significant effects on other genes as reflected in the dynamic Bayesian model.

Although it is not possible to separate the effects of SipA, SopA, SopB, SopD and SopE2 in the data presented here, the known direct and indirect effects of SopE2 and SopB provide crucial links between these 11 mechanistic genes and 14 pathways and the differences between the early phase of WT and MUT infection. The role of SopB during *Salmonella* infection is complex. SopB is a phosphoinositol phosphate phosphatase required for activation of Akt, early trafficking of the *Salmonella*-containing vacuoles (SCV), and activates an SH3-containing guanine nucleotide exchange factor (SGEF) which is an exchange factor for RhoG [33], [35], [68] SopE and SopE2 catalyze nucleotide exchange on Rho GTPases including Rac1, Cdc42, and RhoG and activate MAPK and NF-κβ signaling [34]. These roles of SopE2 and SopB during the early stage of *Salmonella* infection explain the presence and absence of a biphasic response in gene expression in WT and MUT infection respectively. Rac1 and MAPK1, link multiple pathways including the Adherens Junction, BRC signaling, CCR3 Signaling in Eosinophils, MAPK Signaling, Regulation of Actin Cytoskeleton, TGF-β Signaling, Toll-like Receptor Signaling, mTOR Signaling and Wnt Signaling pathways. With the addition of Akt2, Apoptosis, ErbB Signaling, and Tight Junction pathways can be added to the model. Calcium Signaling can be added through the effects of *NFKB1* and *NFKB2*. The Phosphatidylinositol Signaling System cannot be directly linked through these genes but SopB can hydrolyze inositol phosphates and phosphoinositides *in vitro* and is required for formation of phosphatidylinositol 3- phosphate on SCVs [29], [33], [35], [69]. Within the Phosphatidylinositol Signaling System, *PIK3C2G*, *PIK3C3*, and *PRKCG* may represent attractive targets for future studies.

## Discussion

Using microarray analysis, we were able to generate a temporal gene expression profile for 12,257 genes concurrently. Analysis of a relatively large number of genes reduces investigator bias and allows the generation of a gene expression model. Selection of the genes printed on the microarray was independent of our expectations about the effects of *Salmonella* infection or the role of SipA, SopA, SopB, SopD or SopE2. We expected that this method would yield a dataset that would include genes with a known role in *Salmonella* infection as well as identification of expression changes in genes whose role in host response was unexpected. We identified alterations in 38 genes whose functions are as yet unknown. Undoubtedly, the function of these transcripts will be better understood as our understanding of the bovine genome continues to improve and may enhance our understanding of host response to *Salmonella* infection. The microarray data presented here were validated by confirmation of a portion of the altered genes using real time PCR as well as consistency of our results with previously published data. The limitations of this approach are that only changes in expression of genes spotted on the array are measured and that the intestinal tissue used is comprised of multiple cell types, such that it is not possible to differentiate gene expression from one cell type, such as PMNs, from another type, such as epithelial cells; however, we have employed laser-capture microdissection in other studies to evaluate specific gene expression in specific intestinal cell types. Additionally, the data from this study are limited to gene transcription and do not describe alterations in protein expression or state of activation or phosphorylation that may affect activity in the pathways discussed. Studies to address these issues are currently underway in our labs. Ideally, unbiased evaluation of gene expression would include expression data for all genes but at the time that this study was performed, deep-sequencing techniques were cost-prohibitive. Upcoming studies are in progress to utilize these methods and to further develop our understanding of bovine host response to *Salmonella*.

Limitations of our study included the use of four animals instead of a larger number of animals. Previous studies in our laboratory indicated that four animals would be sufficient to detect significant differences between wild type *Salmonella* and the *sipA*, *sopABDE2* mutant [32]. Additionally, we wished to limit the number of animals sacrificed for the study and there were financial limitations on the number of animals and number of microarrays that we could perform. Recognizing that low number of replicates is a common problem in microarray studies, techniques to compensate for low replicates were employed. Specifically, an intensity dependent smoothing variance function was used in the Bayesian inference significance tests [49]. This method borrows strength across the large number of genes measured across all time points. The genes were ranked by intensity levels and a new standard deviation computed by finding the mean of the variances of the next 50 higher genes and the next 50 lower genes surrounding the gene of interest. This produced a Bayesian estimator of the standard deviation that is found to be a reliable approach for statistical testing compensating for the low replicates.

The primary result of this work was the establishment of a temporal gene expression profile and development of an *in silico* model for host response to both wild type S. Typhimurium and a Δ*sipA sopABDE2* mutant. SipA, SopA, SopB, SopD, SopE2 were previously demonstrated to have a combined role in the generation of fluid, induction of cytokines and C-X-C chemokines, and PMN recruitment in bovine ligated ileal loops [9], [12], [32], [70]. The major difference between WT and MUT infections was the presence of a biphasic response with increased activity at one hour post infection in the intestinal loops infected with WT. Using dynamic Bayesian network modeling (DBN) in conjunction with data from KEGG, the Gene Ontology and GenBank, patterns of pathway activation were established for both WT and MUT infection. The differences in gene activity seen in the WT but not MUT were studied in a subset of pathways selected from categories of cell mobility, cell communication, cell growth and death, immune system, membrane transport, and signal transduction. These pathways could be linked through the interactions of two *Salmonella* effectors, SopB and SopE2, and the products of three bovine genes, *RAC1*, *MAPK1*, and *AKT2*. Although this subset of interactions does not explain the entire cascade of interactions observed in the data presented herein, they do serve to validate the model and the DBN approach Our original hypothesis and intent was to generate a profile of host gene expression associated with inflammation and diarrhea using two *Salmonella* strains, wild type and a mutant that generates reduced fluid secretion and inflammatory responses during intestinal infection. Although we have generated gene expression profiles for these two strains, we have not correlated activation or repression of these pathways with the production of fluid in the bovine intestine as a measure of diarrhea. *In vitro* studies employing gene silencing are underway to determine the roles of the 49 pathways identified as differing between the WT and MUT conditions.

In addition to the effects on pathway regulation and mechanistic gene identification, this study identified effects on individual genes in response to infection. We identified increased expression of *CXCL9*, *CXCL10*, and *CXCL11*. This is consistent with previous work in both rhesus macaques and mice which demonstrated that IL17, IL12, and CXCL10 are increased in response to wild type *S.* Typhimurium and that this response is ablated in animals with diminished numbers of CD4+ T cells in the intestinal mucosa [71]. Identification of IFNγ as a mechanistic gene in both WT and MUT loops supports a role for T cells and natural killer cells (both of which produce this powerful cytokine) which is independent of SPI-1 secreted effectors. Increased expression of *CXCL9*, *CXCL10*, and *CXCL11* was observed in WT and MUT loops (Table 5). It has been suggested from work in murine cell lines that TLR4 signaling induces expression of Type I Interferons (interferon-α and interferon-β) [72], [73]. Increased expression of CXCL9, CXCL10, and CXCL11 suggests either that IFN-γ induces the expression of these chemokines or that Type I interferons are increased during the first 12 hours of infection in the bovine intestine. Support for a role of Type I interferons includes studies in mice, which demonstrate that IFNαβ are increased by *Salmonella* infection [73], [74]. Additional indirect evidence includes the increased expression of genes such as *MX2* that is induced during viral infections by IFN-αβ [75]. IFN-αβ is critical for the function of natural killer cell activity [76], [77], [78]. Further study is warranted to define the source of expression of these chemokines and their role in recruitment of T cells and natural killer cells to the intestine during *Salmonella* infection.

In conclusion, this report catalogued the temporal pattern of intestinal gene expression in response to wild type S. Typhimurium in a natural *Salmonella* host, the calf, in response to *S.* Typhimurium infection. We have identified a temporal gene expression pattern comparing wild type *S.* Typhimurium and a strain attenuated for production of diarrhea and inflammation, and we have catalogued the differences observed due to the combined effects of SipA and SopABDE2 on host gene expression. Pathway analysis and the construction of a system level genetic interaction model of the host response have improved our ability to identify important pathways, genetic mechanisms, and novel genes previously not associated with *S.* Typhimurium infection. Further, since the genetic model defined by a dynamic Bayesian network can be trained and used for modeling the effects of silencing selected genes and inferring the possible host response, it will be an invaluable tool for: 1) selecting potential intervening gene targets; 2) guiding future experiments for validating host response models; and 3) identifying dynamic gene expression patterns for early diagnostics.

## Supporting Information

Table S1 ***z*** **-score for All Genes.** The *z*-scores for the individual comparison of gene expression between wild type inoculated loops and control loops, mutant inoculated loops and control loops, and between wild type inoculated and mutant inoculated loops for each gene represented on the microarray are provided. WT = Comparison of gene expression in wild type inoculated loop versus uninfected control loops. MUT = Comparison of gene expression in *ΔsipA*, *sopABDE2* mutant inoculated loops versus uninfected control loops. WvM = Comparison between wild type inoculated loops and *ΔsipA*, *sopABDE2* mutant inoculated loops.
(XLSX)

Table S2 **Fold-Change for All Genes.** The fold-change in gene expression between wild type inoculated loops and control loops, mutant inoculated loops and control loops, and between wild type inoculated and mutant inoculated loops for each gene represented on the microarray are provided. WT = Comparison of gene expression in wild type inoculated loop versus uninfected control loops. MUT = Comparison of gene expression in *ΔsipA*, *sopABDE2* mutant inoculated loops versus uninfected control loops. WvM = Comparison between wild type inoculated loops with *ΔsipA*, *sopABDE2* mutant inoculated loops.
(XLSX)

Table S3 **Detailed gene list for** **Table 2** **Summary of significant genes determined by** ***t*** **test using cutoff** ***p*** **value ≤0.05 and |fold-change| ≥1.5 in response to infection with wild type** ***Salmonella*** **Typhimurium or an isogenic Δ** ***sipA*** **,** ***sopABDE2*** **mutant.** UI = Uninfected control loops inoculated with Luria-Bertani broth. WT = Wild type inoculated loop versus uninfected control loops. MUT = *ΔsipA*, *sopABDE2* mutant inoculated loops versus uninfected control loops.
(XLSX)

Table S4 **Detailed gene list for** **Table 3** **Summary of significant genes determined by Bayesian inference** ***z*** **-score using cutoff |** ***z*** **-score| ≥1.96 and |fold-change| ≥1.5 in response to infection with wild type** ***Salmonella*** **Typhimurium or an isogenic Δ** ***sipA*** **,** ***sopABDE2*** **mutant. UI = Uninfected control loops inoculated with Luria-Bertani broth.** WT = Wild type inoculated loop versus uninfected control loops. MUT = *ΔsipA*, *sopABDE2* mutant inoculated loops versus uninfected control loops.
(XLSX)

Table S5 **Detailed gene list for** **Table 4** **Summary of significantly altered genes that overlap between the two statistical methods (** **Table 2** **and** **3** **).** Genes with significant *z*-score changes and significant *p* value and 1.5-fold-change in response to infection with wild type *Salmonella* Typhimurium or an isogenic Δ*sipA*, *sopABDE2* mutant. UI = Uninfected control loops inoculated with Luria-Bertani broth. WT = Wild type inoculated loop versus uninfected control loops. For genes to be on this list, they had to be found significant based on both statistical analysis methods (*t* test and Bayesian statistical inference).
(XLSX)

Table S6 ***z*** **-score of pathways that differed significantly between loops inoculated with wild type** ***Salmonella*** **Typhimurium or an isogenic Δ** ***sipA*** **,** ***sopABDE2*** **mutant over time.** Pathways are based on Kyoto Encyclopedia of Genes and Genomes descriptions and pathway numbers. Symbol_Count refers to the total number of gene symbols present in a pathway. Observed_Count refers to the number of genes that were observed to be significantly altered at a minimum of one time point. Probe_Count refers to the number of spots on the array that were significantly altered at a minimum of one time point (time in minutes).
(XLSX)

Table S7 **Pathways associated with key mechanistic genes. Key mechanistic genes (determined by Bayesian z-score) from selected pathways identified by dynamic Bayesian Modeling.** Pathways are based on Kyoto Encyclopedia of Genes and Genomes descriptions and pathway numbers.
(XLSX)

File S1 **Description of the methods used in the integrated platform termed the BioSignature Discovery System (BioSignatureDS™) (Seralogix, LLC, Austin, TX).**
(DOC)

## References

[1] (2008). Preliminary FoodNet data on the incidence of infection with pathogens transmitted commonly through food–10 states, 2007.. Centers for Disease Control and Prevention MMWR, Morb Mortal Wkly Rep.

[2] (2010). Preliminary FoodNet data on the incidence of infection with pathogens transmitted commonly through food - 10 states, 2009.. Centers for Disease Control and Prevention MMWR, Morb Mortal Wkly Rep.

[3] Majowicz SE, Musto J, Scallan E, Angulo FJ, Kirk M (2010). The global burden of nontyphoidal *Salmonella* gastroenteritis.. Clin Infect Dis.

[4] Gordon MA, Graham SM (2008). Invasive salmonellosis in Malawi.. J Infect Dev Ctries.

[5] Gordon MA, Graham SM, Walsh AL, Wilson L, Phiri A (2008). Epidemics of invasive *Salmonella enterica* Serovar Enteritidis and *S. enterica* Serovar Typhimurium infection associated with multidrug resistance among adults and children in Malawi.. Clin Infect Dis.

[6] Brent AJ, Oundo JO, Mwangi I, Ochola L, Lowe B (2006). *Salmonella* bacteremia in Kenyan children.. Pediatr Infect Dis J.

[7] Santos RL, Tsolis RM, Baumler AJ, Adams LG (2003). Pathogenesis of *Salmonella*-induced enteritis.. Braz J Med Biol Res.

[8] Zhang S, Kingsley RA, Santos RL, Andrews-Polymenis H, Raffatellu M (2003). Molecular pathogenesis of *Salmonella enterica* serotype *typhimurium*-induced diarrhea.. Infect Immun.

[9] Santos RL, Zhang S, Tsolis RM, Baumler AJ, Adams LG (2002). Morphologic and molecular characterization of *Salmonella typhimurium* infection in neonatal calves.. Vet Pathol.

[10] Santos RL, Tsolis RM, Baumler AJ, Adams LG (2002). Hematologic and serum biochemical changes in *Salmonella* ser Typhimurium-infected calves.. Am J Vet Res.

[11] Santos RL, Schoffelmeer JA, Tsolis RM, Gutierrez-Pabello JA, Baumler AJ (2002). *Salmonella* serotype Typhimurium infection of bovine Peyer's patches down-regulates plasma membrane calcium-transporting ATPase expression.. J Infect Dis.

[12] Zhang S, Santos RL, Tsolis RM, Stender S, Hardt WD (2002). The *Salmonella enterica* serotype *typhimurium* effector proteins SipA, SopA, SopB, SopD, and SopE2 act in concert to induce diarrhea in calves.. Infect Immun.

[13] Takeuchi A (1972). [Electron microscopic observation on the process of microbial invasion into intestinal epithelium].. Nippon Ishikai Zasshi.

[14] Carter PB, Collins FM (1974). The route of enteric infection in normal mice.. J Exp Med.

[15] Galan JE, Ginocchio C, Costeas P (1992). Molecular and functional characterization of the *Salmonella* invasion gene *invA*: homology of InvA to members of a new protein family.. J Bacteriol.

[16] Galan JE, Curtiss R (1989). Cloning and molecular characterization of genes whose products allow *Salmonella typhimurium* to penetrate tissue culture cells.. Proc Natl Acad Sci U S A.

[17] Behlau I, Miller SI (1993). A PhoP-repressed gene promotes *Salmonella typhimurium* invasion of epithelial cells.. J Bacteriol.

[18] Mills DM, Bajaj V, Lee CA (1995). A 40 kb chromosomal fragment encoding *Salmonella typhimurium* invasion genes is absent from the corresponding region of the *Escherichia coli* K-12 chromosome.. Mol Microbiol.

[19] Jones BD, Falkow S (1994). Identification and characterization of a *Salmonella typhimurium* oxygen-regulated gene required for bacterial internalization.. Infect Immun.

[20] Pace J, Hayman MJ, Galan JE (1993). Signal transduction and invasion of epithelial cells by *S. typhimurium*.. Cell.

[21] Chen LM, Hobbie S, Galan JE (1996). Requirement of CDC42 for *Salmonella*-induced cytoskeletal and nuclear responses.. Science.

[22] Francis CL, Starnbach MN, Falkow S (1992). Morphological and cytoskeletal changes in epithelial cells occur immediately upon interaction with *Salmonella typhimurium* grown under low-oxygen conditions.. Mol Microbiol.

[23] Kaniga K, Tucker S, Trollinger D, Galan JE (1995). Homologs of the *Shigella* IpaB and IpaC invasins are required for *Salmonella typhimurium* entry into cultured epithelial cells.. J Bacteriol.

[24] Collazo CM, Galan JE (1997). The invasion-associated type III system of *Salmonella typhimurium* directs the translocation of Sip proteins into the host cell.. Mol Microbiol.

[25] Fu Y, Galan JE (1998). The *Salmonella typhimurium* tyrosine phosphatase SptP is translocated into host cells and disrupts the actin cytoskeleton.. Mol Microbiol.

[26] Hardt WD, Urlaub H, Galan JE (1998). A substrate of the centisome 63 type III protein secretion system of *Salmonella typhimurium* is encoded by a cryptic bacteriophage.. Proc Natl Acad Sci U S A.

[27] Kubori T, Matsushima Y, Nakamura D, Uralil J, Lara-Tejero M (1998). Supramolecular structure of the *Salmonella typhimurium* type III protein secretion system.. Science.

[28] Hardt WD, Chen LM, Schuebel KE, Bustelo XR, Galan JE (1998). *S. typhimurium* encodes an activator of Rho GTPases that induces membrane ruffling and nuclear responses in host cells.. Cell.

[29] Norris FA, Wilson MP, Wallis TS, Galyov EE, Majerus PW (1998). SopB, a protein required for virulence of *Salmonella dublin*, is an inositol phosphate phosphatase.. Proc Natl Acad Sci U S A.

[30] Zhou D, Mooseker MS, Galan JE (1999). An invasion-associated *Salmonella* protein modulates the actin-bundling activity of plastin.. Proc Natl Acad Sci U S A.

[31] Zhou D, Mooseker MS, Galan JE (1999). Role of the *S. typhimurium* actin-binding protein SipA in bacterial internalization.. Science.

[32] Zhang S, Adams LG, Nunes J, Khare S, Tsolis RM (2003). Secreted effector proteins of *Salmonella enterica* serotype *typhimurium* elicit host-specific chemokine profiles in animal models of typhoid fever and enterocolitis.. Infect Immun.

[33] Hernandez LD, Hueffer K, Wenk MR, Galan JE (2004). *Salmonella* modulates vesicular traffic by altering phosphoinositide metabolism.. Science.

[34] Bruno VM, Hannemann S, Lara-Tejero M, Flavell RA, Kleinstein SH (2009). *Salmonella* Typhimurium type III secretion effectors stimulate innate immune responses in cultured epithelial cells.. PLoS Pathog.

[35] Mallo GV, Espina M, Smith AC, Terebiznik MR, Aleman A (2008). SopB promotes phosphatidylinositol 3-phosphate formation on *Salmonella* vacuoles by recruiting Rab5 and Vps34.. J Cell Biol.

[36] Patel JC, Galan JE (2006). Differential activation and function of Rho GTPases during *Salmonella*-host cell interactions.. J Cell Biol.

[37] Bakowski MA, Cirulis JT, Brown NF, Finlay BB, Brumell JH (2007). SopD acts cooperatively with SopB during *Salmonella enterica* serovar Typhimurium invasion.. Cell Microbiol.

[38] Zhang Y, Higashide WM, McCormick BA, Chen J, Zhou D (2006). The inflammation-associated *Salmonella* SopA is a HECT-like E3 ubiquitin ligase.. Mol Microbiol.

[39] Lilic M, Galkin VE, Orlova A, VanLoock MS, Egelman EH (2003). *Salmonella* SipA polymerizes actin by stapling filaments with nonglobular protein arms.. Science.

[40] Schlumberger MC, Friebel A, Buchwald G, Scheffzek K, Wittinghofer A (2003). Amino acids of the bacterial toxin SopE involved in G nucleotide exchange on Cdc42.. J Biol Chem.

[41] Santos RL, Tsolis RM, Zhang S, Ficht TA, Baumler AJ (2001). *Salmonella*-induced cell death is not required for enteritis in calves.. Infect Immun.

[42] Weil MR, Macatee T, Garner HR (2002). Toward a universal standard: comparing two methods for standardizing spotted microarray data.. Biotechniques.

[43] Dudley AM, Aach J, Steffen MA, Church GM (2002). Measuring absolute expression with microarrays with a calibrated reference sample and an extended signal intensity range.. Proc Natl Acad Sci U S A.

[44] Cronin M, Ghosh K, Sistare F, Quackenbush J, Vilker V (2004). Universal RNA reference materials for gene expression.. Clin Chem.

[45] Kim H, Zhao B, Snesrud EC, Haas BJ, Town CD (2002). Use of RNA and genomic DNA references for inferred comparisons in DNA microarray analyses.. Biotechniques.

[46] Rossetti CA, Galindo CL, Everts RE, Lewin HA, Garner HR (2011). Comparative analysis of the early transcriptome of *Brucella abortus*–infected monocyte-derived macrophages from cattle naturally resistant or susceptible to brucellosis.. Res Vet Sci.

[47] Everts RE, Band MR, Liu ZL, Kumar CG, Liu L (2005). A 7872 cDNA microarray and its use in bovine functional genomics.. Vet Immunol Immunopathol.

[48] Loor JJ, Everts RE, Bionaz M, Dann HM, Morin DE (2007). Nutrition-induced ketosis alters metabolic and signaling gene networks in liver of periparturient dairy cows.. Physiol Genomics.

[49] Baldi P, Long AD (2001). A Bayesian framework for the analysis of microarray expression data: regularized t -test and statistical inferences of gene changes.. Bioinformatics.

[50] Livak KJ, Schmittgen TD (2001). Analysis of relative gene expression data using real-time quantitative PCR and the 2(−ΔΔ C_(T)_) Method.. Methods.

[51] Smyth GK (2004). Linear models and empirical Bayes methods for assessing differential expression in microarray experiments.. Statistical Applications in Genetics and Moelcular Biology.

[52] Raffatellu M, Santos RL, Chessa D, Wilson RP, Winter SE (2007). The capsule encoding the viaB locus reduces interleukin-17 expression and mucosal innate responses in the bovine intestinal mucosa during infection with *Salmonella enterica* serotype Typhi.. Infect Immun.

[53] Winter SE, Thiennimitr P, Nuccio SP, Haneda T, Winter MG (2009). Contribution of flagellin pattern recognition to intestinal inflammation during *Salmonella enterica* serotype Typhimurium infection.. Infect Immun.

[54] Troyanskaya OG, Dolinski K, Owen AB, Altman RB, Botstein D (2003). A Bayesian framework for combining heterogeneous data sources for gene function prediction (in *Saccharomyces cerevisiae*).. Proc Natl Acad Sci U S A.

[55] Needham CJ, Bradford JR, Bulpitt AJ, Westhead DR (2007). A primer on learning in Bayesian networks for computational biology.. PLoS Comput Biol.

[56] Murphy K, Mian S (1999). Modeling gene expression data using dynamic Bayesian networks.. University of California.

[57] Scharpf RB, Tjelmeland H, Parmigiani G, Nobel AB (2009). A Bayesian model for cross-study differential gene expression.. J Am Stat Assoc.

[58] Sachs K, Perez O, Pe'er D, Lauffenburger DA, Nolan GP (2005). Causal protein-signaling networks derived from multiparameter single-cell data.. Science.

[59] Hecker M, Lambeck S, Toepfer S, van Someren E, Guthke R (2009). Gene regulatory network inference: data integration in dynamic models-a review.. Biosystems.

[60] Friedman N, Linial M, Nachman I, Pe'er D (2000). Using Bayesian networks to analyze expression data.. J Comput Biol.

[61] (2011). http://www.kegg.com.

[62] Kanehisa M, Goto S (2000). KEGG: Kyoto Encyclopedia of Genes and Genomes.. Nucleic Acids Research.

[63] Friedman N, Geiger D, Goldszmidt M (1997). Bayesian Network Classifiers.. Machine Learning.

[64] Guiney DG (2005). The role of host cell death in *Salmonella* infections.. Curr Top Microbiol Immunol.

[65] Knodler LA, Finlay BB (2001). *Salmonella* and apoptosis: to live or let die?. Microbes Infect.

[66] Gerold G, Zychlinsky A, de Diego JL (2007). What is the role of Toll-like receptors in bacterial infections?. Semin Immunol.

[67] Delbridge LM, O'Riordan MX (2007). Innate recognition of intracellular bacteria.. Curr Opin Immunol.

[68] Steele-Mortimer O, Knodler LA, Marcus SL, Scheid MP, Goh B (2000). Activation of Akt/protein kinase B in epithelial cells by the *Salmonella typhimurium* effector sigD.. J Biol Chem.

[69] Marcus SL, Wenk MR, Steele-Mortimer O, Finlay BB (2001). A synaptojanin-homologous region of *Salmonella typhimurium* SigD is essential for inositol phosphatase activity and Akt activation.. FEBS Lett.

[70] Reis BP, Zhang S, Tsolis RM, Baumler AJ, Adams LG (2003). The attenuated sopB mutant of *Salmonella enterica* serovar Typhimurium has the same tissue distribution and host chemokine response as the wild type in bovine Peyer's patches.. Vet Microbiol.

[71] Raffatellu M, Santos RL, Verhoeven DE, George MD, Wilson RP (2008). Simian immunodeficiency virus-induced mucosal interleukin-17 deficiency promotes *Salmonella* dissemination from the gut.. Nat Med.

[72] Zhang Y, Gaekwad J, Wolfert MA, Boons GJ (2007). Modulation of innate immune responses with synthetic lipid A derivatives.. J Am Chem Soc.

[73] Tam MA, Sundquist M, Wick MJ (2008). MyD88 and IFN-alphabeta differentially control maturation of bystander but not *Salmonella*-associated dendritic cells or CD11cintCD11b+ cells during infection.. Cell Microbiol.

[74] Simon R, Samuel CE (2007). Innate interferon response in macrophage and epithelial cells infected with wild-type compared to DNA adenine methylase and flagellin mutant *Salmonella enterica* serovar Typhimurium.. J Interferon Cytokine Res.

[75] Aebi M, Fah J, Hurt N, Samuel CE, Thomis D (1989). cDNA structures and regulation of two interferon-induced human Mx proteins.. Mol Cell Biol.

[76] Virelizier JL, Gresser I (1978). Role of interferon in the pathogenesis of viral diseases of mice as demonstrated by the use of anti-interferon serum. V. Protective role in mouse hepatitis virus type 3 infection of susceptible and resistant strains of mice.. J Immunol.

[77] Virelizier JL, Griscelli C (1981). [Selective defect on interferon secretion associated with impaired natural killing activity (author's transl)].. Arch Fr Pediatr.

[78] Lipinski M, Virelizier JL, Tursz T, Griscelli C (1980). Natural killer and killer cell activities in patients with primary immunodeficiencies or defects in immune interferon production.. Eur J Immunol.

[79] Rossetti CA, Galindo CL, Everts RE, Lewin HA, Garner HR (2011). Comparative analysis of the early transcriptome of *Brucella abortus* - Infected monocyte-derived macrophages from cattle naturally resistant or susceptible to brucellosis.. Res Vet Sci.

