# Supplementary material for: Role of SPI-1 Secreted Effectors in Acute Bovine Response to Salmonella enterica Serovar Typhimurium: A Systems Biology Analysis Approach
Source: PLoS One. 2011 Nov 11;6(11):e26869. doi: 10.1371/journal.pone.0026869 (PMC3214023; doi:10.1371/journal.pone.0026869)
Supplement: File S1 — Description of the methods used in the integrated platform termed the BioSignature Discovery System (BioSignatureDS™) (Seralogix, LLC, Austin, TX). (DOC) [file pone.0026869.s008.doc]

**Supporting File S1 Description of BioSignatureDS Computational Methods**

The core computational tools in BioSignatureDS (<http://www.seralogix.com/>, Seralogix, LLC, Austin, TX) are based on the probabilistic power of Dynamic Bayesian Networks (DBN), an advanced form of machine learning and pattern recognition developed over the past several years with funding from the National Institutes of Health. At present, there are different computational and statistical approaches to the “omics” data analysis and modeling, including clustering (or naïve correlation), Boolean models, differential equations, relevance networks, graphical Gaussian nets, genetic algorithms, neural networks, static Bayesian network and dynamic models [1]. However, statistical methods such as clustering or ANOVA does not allow for the analysis and inference of system-level dependencies. Owing to their probabilistic nature, Bayesian networks have a number of advantages over models like Boolean nets, differential equations, relevance or graphic Gaussian nets. These advantages include the modeling of uncertainty, handling of hidden variables, automated learning, and inference of regulatory modules. Moreover, Bayesian networks are biologically interpretable and can be scored rigorously against observation data, rendering them preferable to complex non-linear models (e.g. neural nets). A large body of work has successfully applied Bayesian networks in various learning and interpretations of biological systems [2-7]. Differing from most of the previous work, which only consider the static gene or protein relationships, BioSignatureDS applies Dynamic Bayesian networks that support time-series data of unequal time sampling intervals and enable the modeling of complex temporal processes and feedback loops [8]. Moreover, the Dynamic Bayesian networks are developed for continuous variables, thereby avoiding the information loss associated with discretization. In addition, the Bayesian tools utilize a combination of data and prior biological knowledge to learn interaction network models which best explain the observed gene/protein dependencies given the current state of knowledge embodied by these inputs.

Through the DBN-based analysis and modeling, one key innovation of the BioSignatureDS is the identification of groups and individual genes/proteins that as a whole represent the perturbation in a pathway, pathway subnet, or biological process over time. This technique is named Dynamic Bayesian Gene Group Activation (**DBGGA**) (Seralogix, LLC, Austin, TX). DBGGA examines and ranks groups of interrelated genes across all time points in lieu of just one gene in a single time point, in a computational methodology that determines the differences and commonalities between experimental conditions and disease states. DBGGA also enable us to determine which pathways are most perturbed in one condition relative to another and what genes are the significant sources of the perturbation (such genes are designated as “**candidate mechanistic genes**”) and is described in more detail below. This is a totally unique and innovative application of DBNs for gene discovery analysis.


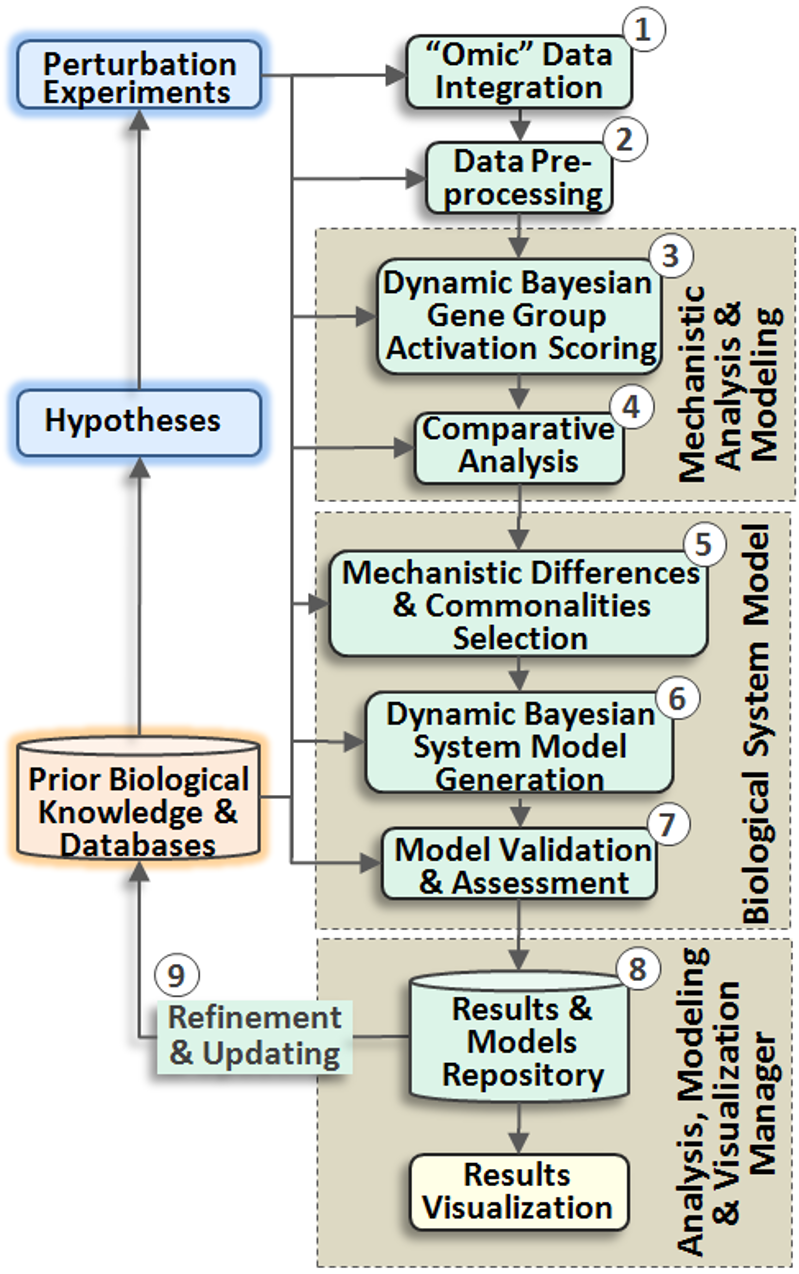


Figure S1. Systems biology cycle depicting the computational pipeline steps and visualization embedded in the BioSignatureDS.

In addition, BioSignatureDS utilizes the significantly perturbed pathways, gene groups and the “candidate mechanistic genes” as building blocks to construct a plausible network model of the disease/condition. Encapsulating global time-course patterns and multi-conditional behaviors of a large group of genes/proteins, the systems level model has great discriminating power even when the effects of individual genes are small. Thus, the disease models can be used for more efficient comparative modeling, pattern recognition (diagnostics) and simulations for “what-if” analyses (prognostics).

The BioSignatureDS consists of integrated set of custom developed software tools having an assortment of advanced computational capabilities and relational database storage. The platform (developed in Java) is designed to implement a computational pipeline and to provide extensible, flexible and efficient access to and visualization of related data at various stages of the analysis/modeling process. BioSignatureDS is designed to support the systems biology cycle that involves the iterative process of experiment-modeling-refinement as depicted in Figure S1. Embodied in this cycle is the computational pipeline whose main steps are labeled 1-9 in Figure S1. The platform provides for all the necessary data management and computational steps in a nearly seamless data workflow enabling high throughput data analysis/modeling. At major steps in the pipeline, the dynamic Bayesian network (DBN) modeling engine is used to score and select mechanistically significant regulatory points and modules (Step 3) and to construct system level disease models for simulation, inference and pattern recognition (Step 6). Once the analysis/modeling is completed for the experimental conditions being considered (Step 7), the results are stored in a repository/database (Step 8) for access by research scientists via the Analysis, Modeling and Visualization Manager (AMVIZmanager™) (Seralogix, LLC, Austin, TX) described in more detail below. Analysis results are utilized by the research scientists to generate new hypotheses and to refine and guide the design of future experiments. The capture of new biological discoveries is preserved by updating the Prior Biological Knowledge Database for reuse in future analyses and model refinements (Step 9).

***“Omics” data Integration*.** A relational database was constructed to store and organize user imported experimental data and prior biological knowledge (i.e., pathways, interactions, etc.). The data ranges from time-resolved genome-wide gene expression arrays and protein abundance measurements to physiological responses and symptomic observations. Prior biological knowledge databases that reside on servers and updated routinely include KEGG, REACTOME, JASPAR, Genbank, BIOBASE, Gene Ontology, protein-protein interactome (NCBI), gene-chromosome mapping and others. The BioSignatureDS unifies synonyms based on standard ontologies (GO, NCBI) and utilized a comprehensive ID system to keep the semantics consistent between the data entities from the different resources. A set of custom developed utility tools supports importing and updating prior biological knowledge for all the data types employed in the analysis and modeling process such as the latest genome annotations from any available species or the custom creation of a hypothetical pathway (Figure S1. Step 1).

***Data Preprocessing***. A Java-based management tool, *Xmanager™* (Seralogix, LLC, Austin, TX), was specifically designed for importing, preprocessing, and managing multi-conditional experimental data (Figure S1 Step 2). *XManager* semi-automatically imports and mines the time-course experimental gene array, protein and physiological experimental data into a relational database. All entries are annotated according to the unified symbol and ID system during the process. *Xmanager* is suitable for many data formats including GenePix Results, Affymetrix (GCOS), Codelink, Agilent or proteomics data from Mass Spectrometry or Next Gen Sequencing; and is easily extensible to include any other measurement format/platform. The preprocessing engages a series of steps including normalization (global scaling, mean-, median-, Lowess-normalization), quality assessment and filtering (coefficient of variance, signal-to-noise ratio, background randomness, signal correlation), channel/array merging and classical statistic analyses (z-test, t-test, Bayesian statistics, fold, boxplot etc.).

***Mechanistic Analysis & Modeling.*** Following the data integration and preprocessing steps in the computational workflow, BioSignatureDS executes the Mechanistic Analysis and Modeling steps (Figure S1. Steps 3-4) to analyze, discern, and model the unique differences and similarities over the consolidated multi-condition observations. Central to the analysis and modeling is the application of Dynamic Bayesian networks (DBNs). A technique was developed for identifying groups of genes that as a whole represent the activation/inactivation of metabolic/signaling pathways, pathway subnets, biological processes, and genes over time. This technique is extremely important due to lack of sample measurement repetitions, the variable span of time between measurements, and the resulting expression level variability commonly observed between experiments. This technique termed Dynamic Bayesian Gene Group Activation (**DBGGA**) allows the user to examine, score, and rank groups of genes across all time points in lieu of just individual genes in a single time slice to determine differences within components (subsystems) of a biological system as a function of the experimental conditions. These components are defined by the prior biological knowledge such as the best known gene/protein relationships involved, for example, in the Toll-like Receptor Pathway or the Carbohydrate Metabolism Pathway, or a group of genes associated with the Gene Ontology term “dorsal/ventral pattern formation”. Hundreds of pathways and thousands of biological processes are routinely analyzed in this fashion to systematically identify the biological system behavior as a function of the different experimental conditions.

*Dynamic Bayesian Network (DBN) Engine.* The underlying DBGGA computational procedures rely on Dynamic Bayesian Networks. A DBN is a directed, acyclic graphical (DAG) model of a stochastic process. The DBN technology has been extensively tested on numerous analysis projects. The DBN consists of time-slices (or time-steps), with each time-slice containing its own variables (e.g. gene/protein nodes). It is defined as the pair (*BN*_0_, *BN*_→_ ) where *BN*_0_ defines the prior or initial state distribution of the state variables *P*(*Z_t=_*_0_) and *BN*_→_ is a two-slice temporal Bayesian network (2TBN) that defines the transition model *P*(*Z_t_*|*Z_t_*_-1_) as follows:


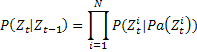


Where
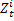
 is the *i*-th node (gene, protein, etc) at time slice *t*.
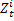
 can be a hidden node, an observation node, or another type of specialized node for classification. *Pa*(
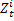
) are the parents of
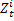
 which can be in the same or previous time-slice. The parameters *θ* defining the conditional probability
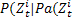
 )) associated with each node variable
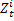
, may then be learned from a training dataset by application of the Expectation-Maximization (EM) algorithm [9]. The EM algorithm determines the parameters
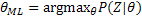
 maximizing the model probability given the training datasets. The training dataset, *D*, is a set of observations (e.g. gene expression, protein levels, etc.) for each time slice *t*, over a total sampling time period. In this way, an unbounded temporal sequence length of *T* time-slices can be modeled using a finite number of parameters. The DBN is realized by “unrolling” the 2TBN until there are *T* time-slices (Figure S2). The joint probability distribution of this DBN is defined by:


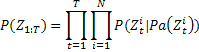


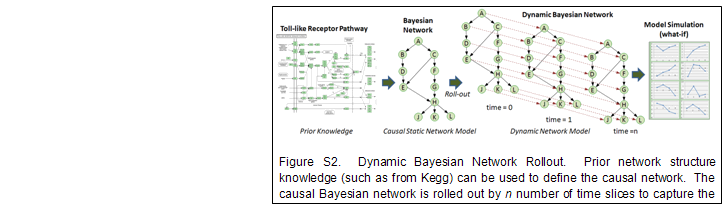


DBN models in BioSignatureDS™ are tailored for the needs of quantitative analysis of time-series data. In particular, the DBN models are built upon treating gene/protein expression values as continuous variables to minimize information loss from the data during model construction. Most prior work in Bayesian-network based modeling has assumed 2- or 3-discrete state distributions for the modeled variables, or in some cases a mixture of discrete nodes with more restricted type of continuous nodes than considered herein [10, 11]. In addition, regularizations (such as Tikhonov regularization [12, 13]) are applied in the DBN training process to minimize overfitting, which is a concern in inferring behaviors of thousands of interlinked genes from a small number of data observations.

*Mechanistic Analysis and Modeling Approach using DBGGA.* The main output of the Mechanistic Analysis and Modeling steps is the comprehensive comparative analysis and modeling of components within a biological system having multiple experimental conditions (e.g. perturbed by external stimuli). The DBN model-based comparisons between several different experimental measurements are automated to score and select genes or groups of genes that are responsible for the activation/inactivation of pathways, pathway subnets, or biological processes over time as identified by the DBGGA procedure. This scoring procedure essentially relies upon prior network structure knowledge and the ability of Bayesian Network models to assess the statistical likelihood of a given set of data values for the variables (i.e., the network nodes). It needs to be emphasized that this likelihood assesses the entire set of values taken as a whole. This is critical, as the statistical relationships/correlations (i.e., weighted directed network edges) represented by the DAG network structure imply that the expected value (e.g., gene expression level) of a given network node (e.g. a gene) depends on the values of the neighboring nodes.

DBGGA scores a given gene or group of genes by evaluating the contribution of the associated data for the gene or group to the Bayesian Network likelihood. This contribution (for a particular time slice *t*) may be measured by the conditional probability
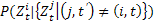
. In practice, we are interested in scoring genes/groups with regard to their contribution in differentiating experiments from controls. Thus, we compute the conditional likelihoods
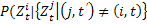
 of both the control-condition and the experimental-condition data with the DBN model parameters optimized for the control-condition. A two sample, un-paired T-test is then used to assign p-values to the hypothesis that the gene group or an individual gene experimental-condition log-likelihoods are significantly lower than their control-condition log-likelihoods as assessed by this control condition DBN model. The lower log-likelihood implies that the experimental condition for the given gene group is divergent (poorer model fit) from the control condition. These p-values may then be converted to z-score equivalents, such that a high z-score indicates a strong contribution to the distinguishing of experiment from control. In some rare cases the experimental data has been observed to have better fitting data to the control model than the control data resulting in a negative z-score. In these cases the z-score is floored to zero since we are only interested in the hypothesis that the experimental data is a worse fit to the control model. Since the log-likelihoods cannot distinguish the direction of perturbation (i.e., up-regulated vs. down-regulated in the case of an individual gene or induced versus suppressed for pathways and GO groups), the last step for computing the final score is to multiply the z-scores by +1 if the average of the experimental condition data is greater than the control condition data and -1 if it is lower. This transformation from likelihood to a z-score is referred to hereafter as the **DBGGA Bayesian Z-score (Bayesian z-score)**. An individual gene that meets a user specified significance threshold is termed a **candidate mechanistic gene**, because this gene contributes significantly to the perturbed state of the pathway in which it is associated.

The DBGGA scoring can be applied at the pathway level which is a group of interconnected genes. The data for all the genes in the pathway from the perturbed experimental condition is scored against the control DBN model as described above. This results in the ability to compare the conditional probability-based score with those of several hundred other pathways that were scored as part of the system’s analysis. Thus, it is possible to rank and select the pathways and/or GO groups that are most perturbed under differing disease conditions. Similarly, individual genes may be scored within the pathway models. Figure S3a illustrates the comparison of pathway scores (as a heatmap) for a set of highly perturbed pathways as they change over time. The example is for the bovine host infected with *Salmonella enterica* Serotype Typhimurium. Figure S3b is the network representation of the Regulation of Actin Cytoskeleton Pathway which was one of the significantly perturbed pathways the heatmap in (a). Figure S3c are the significantly perturbed gene score heatmap for this pathway.

For each experimental condition, the trained network model (such as Figure S3b) can be interrogated for mechanistic genes (meets a Bayesian z-score threshold defined by the user) across all time points and the strength (correlation) of connections between interconnecting genes. Each pathway model can produce a heat map of candidate mechanistic gene scores (at any selected threshold) by time point.


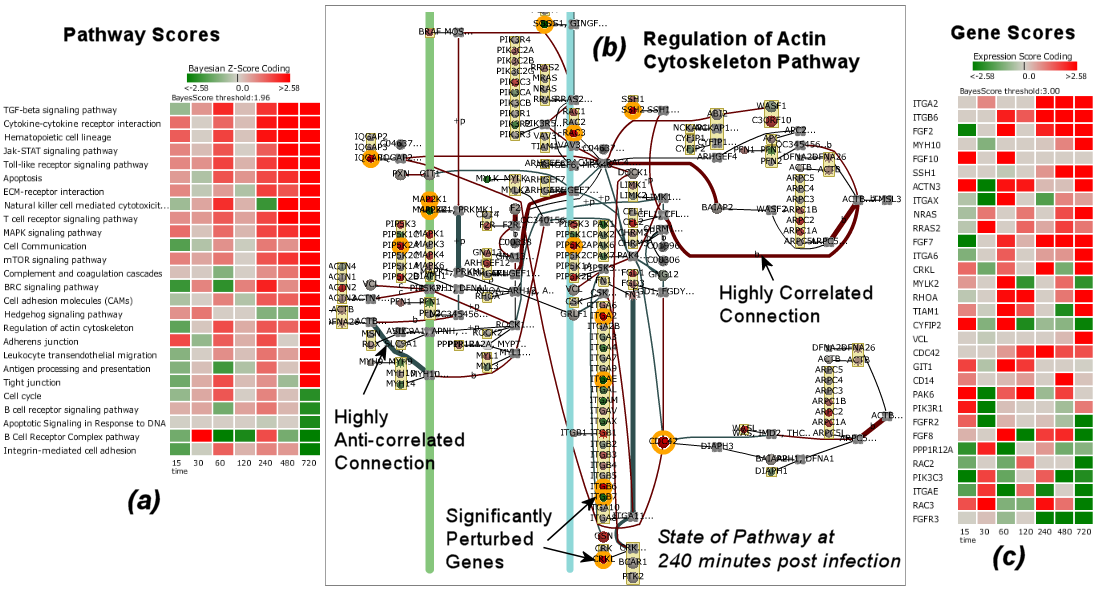


Figure S3. An example pathway analysis found to be highly perturbed. (a) This analysis is from the bovine host infected with *Salmonella enterica* Serotype Typhimurium. Seven time points (15, 30, 60,120, 240, 480, and 720 minutes) were sampled post infection resulting in the pathway scores shown in the heat map. (b) This is an example of Regulation of Actin Cytoskeleton with its state of perturbation shown at 240 minutes post infection. Nodes represent genes, gradients of red indicated up regulation and gradients of green are down regulation. Genes that are highlighted with orange rings are those determined to be significantly perturbed. The size of the arcs connecting genes represents the correlation between genes as determined from our training algorithms. (c) These are the genes for the Actin Cytoskeleton pathway that are considered significantly perturbed over the course of 720 minutes. The heat map makes it easy to see the state of the gene expression at each time point in comparison to others on the pathway.

*Validation of DBGGA*. Significant research has gone into validating the DBGGA technology under the original grants in which this technology was developed (unpublished due to proprietary nature of the technique). Both statistical methods employing synthesized data and real gene expression data were employed. The DBGGA method outperformed traditional methods such as hypergeometric-based enrichment detection techniques (e.g. GeneMerge [14] and GOminer [15]) in determining subtle changes in gene expression levels for groups of interrelated genes. Sensitivity and selectivity for distinguishing perturbed genes while increasing additive white noise also proved more robust than these traditional methods. The identification of significant gene perturbation by DBGGA has been experimentally confirmed by qRT-PCR in several recent studies (unpublished results).

***Disease (System) Model Generation.*** The BioSignatureDS is also employed to integrate the significantly perturbed pathways and gene groups from DBGGA to construct a plausible system level model of the disease (Fig. S1 Steps 5-7). The system model encompasses the whole time-course patterns and multi-conditional behaviors of a large group of genes/proteins. The system (disease) model can be used for more efficient comparative modeling, pattern recognition and simulations supporting “what-if” analyses. The system modeling is based on first merging genes and relationships taken from the top seven scored pathways identified from DBGGA analysis (Fig S1 Step 5) into a composite DBN. The system model for host infected with WT and Δ*sipA, sopABDE2* mutant *S.* Typhimurium is shown in the main text Figure 4. The networks can be used to explore the differences between the WT and mutant conditions and to identify genes that are uniquely governing the host response to pathogen invasion and to identify genes that could be potential targets of therapeutic intervention.

**References for Supplemental File S1**

1. Hecker M, Lambeck S, Toepfer S, Someren Ev, Guthke R: **Gene regulatory network inference: Data integration in dynamic models—A Review**. *BioSystems* 2009, **98**:86–103.

2. Friedman N, Linial M, Nachman I, Pe'er D: **Using Bayesian networks to analyze expression data**. *J Comput Biol* 2000, **7**:601–620.

3. Sachs K, Perez O, Pe’er D, Lauffenburger DA, Nolan GP: **Causal protein-signaling networks derived from multiparameter single-cell data**. *Science* 2005, **308**:523–529.

4. Troyanskaya OG, Dolinski K, Owen AB, Altman RB, Botstein D: **A Bayesian framework for combining heterogeneous data sources for gene function prediction (in Saccharomyces Cerevisiae)**. *Proc Natl Acad Sci U S A* 2003, **100**:8348–8353.

5. Beaumont MA, Rannala B: **The Bayesian revolution in genetics**. *Nat Rev Genet* 2004, **5**:251–261.

6. Heckerman D: **A tutorial on learning with Bayesian networks**. In: *In: Jordan MI, Learning in graphical models.* in Jordan MI, Dordrecht: Kluwer Academic; 1998: 301–354.

7. Imoto S, Higuchi T, Goto T, Tashiro K, Kuhara S, Miyano S: **Combining microarrays and biological knowledge for estimating gene networks via Bayesian networks**. *J Bioinform Comput Biol* 2004:77-98.

8. Needham CJ, Bradford JR, Bulpritt AJ, Westhead DR: **A Primer on Learning in Bayesian Networks for Computational Biology**. *PLoS Comput Biol* 2007, **3**(8).

9. Lauritzen SL: **The EM algorithm for graphical association models with missing data**. *Computational Statistics and Data Analysis* 1995, **19**:191–201.

10. Helman P, Veroff R, Atlas SR, Willman C: **A Bayesian Network Classification Methodology for Gene Expression Data**. *JOURNAL OF COMPUTATIONAL BIOLOGY* 2004, **11**(4):581-615.

11. Friedman N, Nachman I, Peer D: **Learning Bayesian Network Structure from Massive Datasets: The “Sparse Candidate” Algorithm**. *Proc Fifteenth Conference on Uncertainty in Artificial Intelligence (UAI ’99)* 1999:196–205.

12. Tikhonov AN, Arsenin VY: **Solutions of ill posed problems** Washington, D.C.: Winston; 1977.

13. Hoerl AE, Kennard RW: **Ridge Regression: Biased Estimation for Nonorthogonal Problems**. *Technometrics* 1970, **42**(1):80-86.

14. Castillo-Davis CI, Hartl DL: **GeneMerge-post-genomic analysis, data, and hypothesis testing**. *Bioinformatics* 2003, **19**:891-892.

15. B. Zeeberg B, Feng W, Wang G, Wang M, Fojo A, Sunshine M, Sudarshan Narasimhan, Kane D, Reinhold W, Lababidi S, Bussey K, Riss J *et al*: **GoMiner: A Resource for Biological Interpretation of Genomic and Proteomic Data**. *Genome Biology* 2003, **4**(4).
